# Applying transcriptomics to studyglycosylation at the cell type level

**DOI:** 10.1016/j.isci.2022.104419

**Published:** 2022-05-18

**Authors:** Leo Alexander Dworkin, Henrik Clausen, Hiren Jitendra Joshi

**Affiliations:** 1 Copenhagen Center for Glycomics, Department of Cellular and Molecular Medicine, Faculty of Health Sciences, University of Copenhagen, Blegdamsvej 3, 2200 Copenhagen N, Denmark

**Keywords:** Molecular biology, Omics, Transcriptomics

## Abstract

The complex multi-step process of glycosylation occurs in a single cell, yet current analytics generally cannot measure the output (the glycome) of a single cell. Here, we addressed this discordance by investigating how single cell RNA-seq data can be used to characterize the state of the glycosylation machinery and metabolic network in a single cell. The metabolic network involves 214 glycosylation and modification enzymes outlined in our previously built atlas of cellular glycosylation pathways. We studied differential mRNA regulation of enzymes at the organ and single cell level, finding that most of the general protein and lipid oligosaccharide scaffolds are produced by enzymes exhibiting limited transcriptional regulation among cells. We predict key enzymes within different glycosylation pathways to be highly transcriptionally regulated as regulatable hotspots of the cellular glycome. We designed the Glycopacity software that enables investigators to extract and interpret glycosylation information from transcriptome data and define hotspots of regulation.

## Highlights

• RNA-seq can provide information on the glycosylation metabolic network state
• It is possible to readout glycosylation capacity from single cell RNA-seq data
• Genes regulating the biosynthesis of common glycan scaffolds show little regulation
• Key enzymes in the glycosylation network are predicted to be regulatable hotspots

## Graphical abstract

**Figure:**
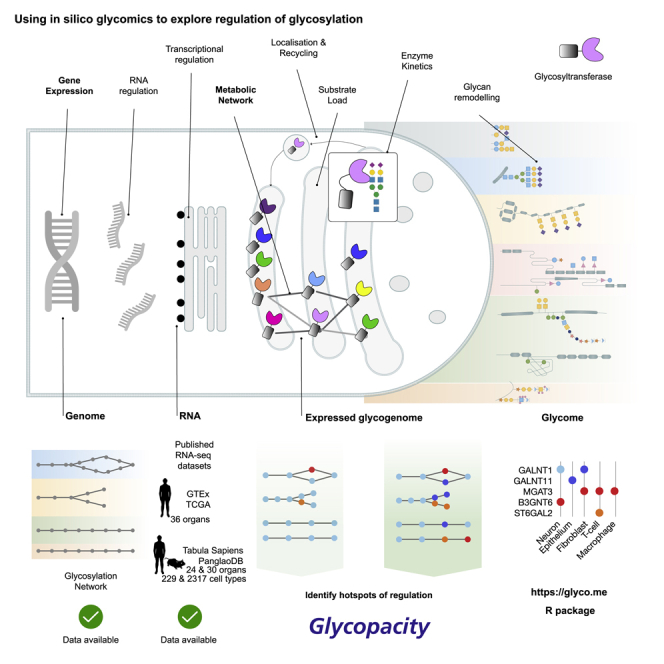

## Introduction

The structural diversity of a cellular glycome is daunting; it comprises glycans found on proteins, proteoglycans, and lipids, as well as free oligosaccharides (Cummings, 2009). Glycosylation is the most abundant and diverse posttranslational modification of proteins (Schjoldager et al., 2020). Most (>85%) proteins trafficking the secretory pathway are glycosylated (Steentoft et al., 2013; Zielinska et al., 2010) and most proteins in the nucleus and cytoplasm undergo O-GlcNAcylation (Hart, 2019), which greatly amplifies the proteome by producing diverse proteoforms with different functional properties and myriad functions (Aebersold et al., 2018; Spiro, 2002; Varki, 2017).

The glycosylation processes of proteins and lipids involve a complex metabolic network of sequential enzymatic steps employing at least 174 glycosyltransferases, 35 sulfotransferases, and 3 epimerases (together, the glycogenes), whose properties to a large extent determine which proteins become glycoproteins, where the glycans are positioned on the proteins, and the structures of the elaborate glycans attached (Schjoldager et al., 2020). Arguably, the principal factor that determines the outcome of the glycosylation network in a cell is the available repertoire of glycosyltransferases. The kinetic properties of glycosyltransferase enzymes are largely equipped with sufficient acceptor and donor substrate preferences, kinetic efficiencies and subcellular localization signals for unsupervised attachment and stepwise assembly of oligosaccharides in the secretory pathway. This has allowed us to assemble a global atlas of cellular glycosylation pathways, as well as assigning glycosyltransferases directing these pathways (Joshi et al., 2018; Narimatsu et al., 2019; Schjoldager et al., 2020). The atlas of glycosylation pathways describes the topology of a generic glycosylation network in a cell. We hypothesize that from cell to cell, differential transcriptional regulation of glycosyltransferases will impact the availability of different pathways in the network to the glycosylation processes and will be a major factor in regulating diversity and shape of the cellular glycome. This is clearly a simplistic view and we already know that many other factors will affect glycosylation, including acceptor substrate and donor sugar availabilities, competition among enzymes, co-factors, pH, chaperones and glycosidases, residence time in the secretory pathway, the compartmental organization of the glycosylation machinery (Moremen et al., 2012; Schjoldager et al., 2020; Welch and Munro, 2019), and more general factors such as cellular stress and e.g., malignant transformation (Moremen et al., 2012).

The glycosylation machinery is organized into not only relatively stringent sets of pathway specific enzymes that coordinate biosynthesis of distinct core glycan structures (of which humans have 17), but also into pathway non-specific enzymes that often share substrates among several pathways and result in generation of common scaffolds and different terminal modifications (Cummings, 2009; Schjoldager et al., 2020). The diversity in the glycome arises from the unique structural scaffolds built within each glycosylation pathway, from elaborations on these scaffolds using repeated common structural motifs, and from diversity in common terminal modifications. Regulation of this diversity relies not only on unique glycosyltransferases directing individual steps in the biosynthesis, but also the use of isoenzymes with partly overlapping and partly unique functions as this provides for options for differential regulation of subtle structural changes in the glycome. The latter group of isoenzymes is still incompletely understood, and these currently pose the main challenge for predicting the cellular glycosylation capacity from expression data of glycogenes (Schjoldager et al., 2020).

Glycosylation takes place in the individual cell, and the glycome by-and-large results directly from the organization of the metabolic glycosylation network in the cell. We cannot see the results of this process in a single cell using current structural analytics, and most of our understanding of the glycome stems from direct structural analysis of heterogeneous cell or tissue preparations, and thus informs us of an averaged snapshot of the glycan structures found in many cells (Čaval et al., 2021; Khoo, 2019; Lageveen-Kammeijer et al., 2021; Levery et al., 2015; Thaysen-Andersen and Packer, 2014). An exception to this is the use of glycan-binding probes (lectins, antibodies, glycan-binding proteins) for binding to cells and tissues, and this approach, although limited by the number of specific probes to the majority of glycan structures (Bojar et al., 2021), suggests that expression of select glycan structures can be highly regulated on cells during the cell cycle and during cellular maturation and differentiation (Dabelsteen et al., 1991; Park et al., 2021; Thomas, 1971). Today, the best chances to circumvent the heterogeneity of cell populations come from technologies that have already broken the single cell barrier. Single cell transcriptomics (and soon proteomics) offers a methodology to capture the state of the glycosylation machinery, i.e., the expression levels of the enzymes involved, at the single cell level, which can be used to predict the cellular glycosylation capacity and glycome outcome.

Thus, the rapidly emerging high quality single cell RNA-seq transcriptomic data offers a unique opportunity for the glycomics field to probe the regulation of glycosylation enzymes at the single cell level. Key to utilizing this information is the ability by which we can transform it to provide useful information about the glycosylation outcome, i.e., the glycome. Single cell RNA-seq experiments typically capture the most highly expressed genes, and so technical dropouts are more biased toward lowly expressed genes (Hicks et al., 2018) relative to traditional bulk sequencing (Svensson et al., 2017). These challenges make it difficult to ensure that the complete glycogene repertoire can be extracted and analyzed from any given single cell dataset. Here, we hypothesized that we can quantify transferase levels at the cell type level by performing an *in-silico* amplification of signal from clusters of single cells, rather than relying on quantifying transferase levels in each cell directly. This approach enabled us to map the broad contours of the landscape of transcriptional variation for all glycogenes from the organ to single cell level. The map reveals specific glycosylation pathways that are present in defined cell types and enables estimation of the ranges of expression for glycogenes in healthy organs and cells, which we then use to predict key hotspots in regulation of cellular glycosylation and the glycome. We used the data to develop a software package Glycopacity, which we have also made available as a web tool online at https://glyco.me.

## Results

### Patterns of regulation at the organ level

**Figure 1:**
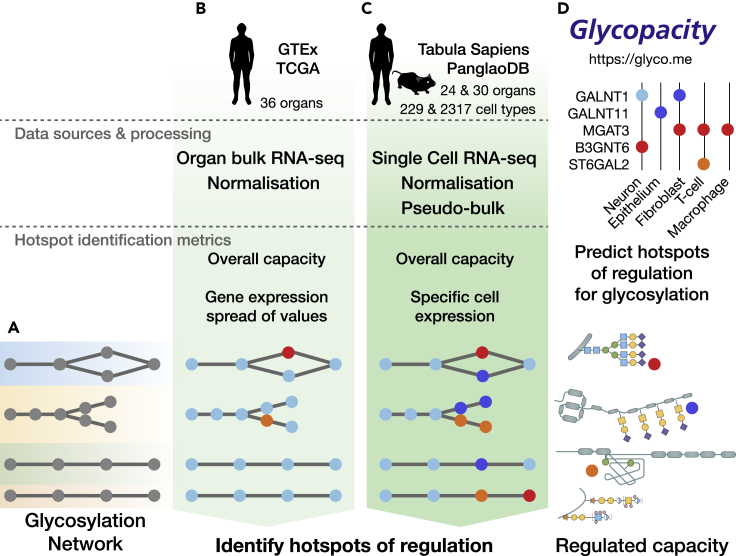
Development of a tool to identify hotspots of regulation on the metabolic glycosylation network (A) We have over recent years been curating knowledge and mapping out the network of genes that control glycosylation in cells (the rainbow representation of glycosylation pathways, Figure S1). The availability and activity of glycogenes that make up glycosylation pathways within this network help to define the capacity for glycosylation that each cell can perform. Individual pathways in the network are regulated differently, and we used data mining of mRNA transcriptional data to better understand how this network is differentially regulated between organs and cells. (B) Using organ-level bulk RNA-seq data, we estimated overall glycosylation capacity, and the spread of gene expression values to identify hotspots of regulation of the glycogenome. (C) We further refined these hotspots of regulation by increasing the granularity of regulation from the organ level to the single cell level. Using single cell RNA-seq data, we performed an analysis that enabled predictions of the ubiquitous and regulated parts of the glycome from over 200 different cell types from human organs and tissues. (D) We have created a software package to make these tools for predicting the glycosylation capacity from bulk and single cell RNA-seq data accessible to the research community as an R package and a website at https://glyco.me. The software package enables generation of heatmaps predicting hotspots of regulation for glycosylation, and can predict not only the overall capacity for glycosylation from input expression data, but what part of this capacity is likely regulated. See also Figure S1, Tables S1 and S2.

We first turned to organ-level bulk RNA-seq data to identify hotspots of regulation in the glycosylation network (Figure 1A), building on our previous analysis that applied a simplistic metric (Tau) to identify hotspots (Joshi et al., 2018). For this new analysis we aimed to understand the behavior of two aspects of regulation between organs: 1) the overall capacity for glycosylation inferred by all detectable/active mRNA transcripts of glycogenes (i.e., the glycosyltransferase and glycan sulfotransferase genes) and 2) the baseline and dynamics of transcripts for individual glycogenes among organs. We hypothesize that the latter will provide for useful reference ranges of transcript quantitation for the single cell data.

#### The rainbow depiction of glycosylation pathways

We have over recent years refined an atlas of glycosylation pathways (Schjoldager et al., 2020) that organises the glycogenome (currently defined as a set of genes primarily comprising 174 genes encoding glycosyltransferases, and 35 encoding sulfotransferases) into 17 distinct glycosylation pathways, and three further groups that do not belong to any specific pathway (non-specific elongation, capping and sulfation). This atlas is represented in the rainbow depiction of basic glycosylation steps, covering all known glycosidic linkages known to be formed in human cells (Figure S1). The rainbow depiction distinguishes between glycogenes that serve functions in specific glycosylation pathways and those that serve multiple pathways and hence are designated pathway non-specific. This provides a framework for relatively reliable prediction of the glycosylation outcome for 2/3 of the glycogenes, whereas the residual 1/3 comprising pathway non-specific glycogenes enable predictions of linkages but not reliable prediction of the types of glycoconjugate involved.

#### Sourcing of organ transcriptomic data

Bulk RNA-seq transcriptomic data (GTEx and TCGA) from human organs were downloaded from the recount2 resource (Collado-Torres et al., 2017), and we retrieved count data covering 36 organs and 10,402 samples (Figure 1B, Samples and organ information listed in Table S1). We normalized the count data using TMM (Robinson et al., 2010), producing counts-per-million (CPM) for each gene and sample, which we then normalized to the average expression level of a panel of housekeeping genes (see STAR Methods). This yielded CPM values that could be compared between organs for 224 glycogenes (which include the glycosyltransferases, sulfotransferases, one chaperone (C1GALT1C1), one hexokinase (POMK), three epimerases, one deacetylase, and nine genes with a glycosyltransferase domain whose activity is currently unknown).

#### Metrics to identify hotspots of regulation of glycosylation

We previously calculated a quantitative measure for the ubiquity of expression of glycosyltransferases using Tau (Joshi et al., 2018). The Tau metric provided for a simplified view into the regulation of glycosyltransferases that could easily identify genes that were highly expressed in few tissues but could not identify which genes were downregulated in only few organs, or report the magnitude of variation in expression for less extreme cases. To remedy this, we chose to calculate two metrics for each glycogene for the normalized data: the first metric is the percentage of organs where the average CPM (overall samples) for each glycogene is above a minimal cut-off. This metric contributes to our understanding of the overall capacity for glycosylation for each organ. The second metric is the inter-quartile range (IQR) of CPMs across all samples for each glycogene, which informs us about the magnitude of variation in mRNA expression between organs.

This allowed us to generate a visual map of the glycosylation capacities. The overall glycosylation capacity for an organ (i.e., the overall repertoire of glycogenes with detectable transcripts) determines if pathways within the glycosylation network are open to be followed or blocked and thus inactive. From the active pathways of the glycosylation network, we can thus predict the glycosylation outcome.

**Figure 2:**
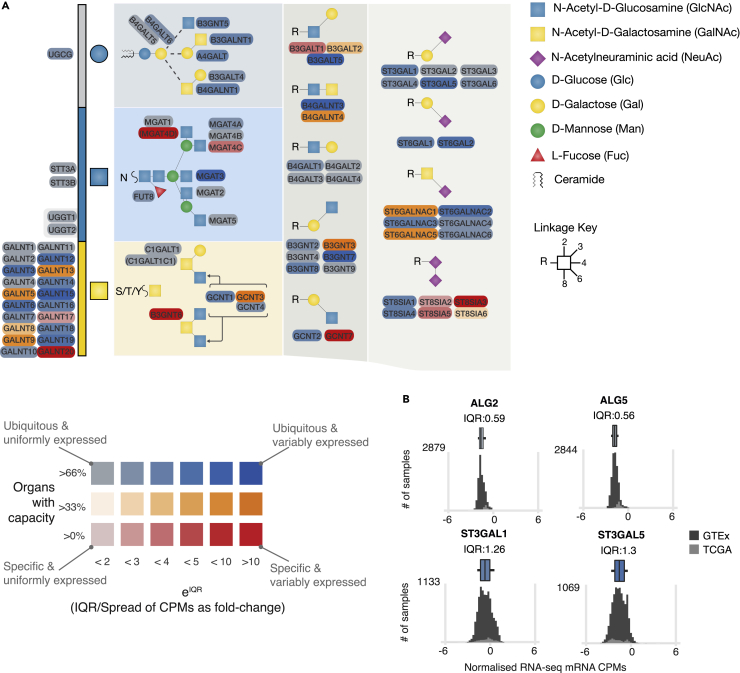
Identification of hotspots of regulation in the glycosylation network from organ-level data (A) Extract of a glycogene based heatmap indicating the hotspots of regulation found on the glycosylation network (Full heatmap available in Figure S2A). The percentage of organs that glycosylation capacity is found in is mapped to the color (blue, orange and red according to increasing organ specificity), whereas the spread of the expression values maps to the saturation of the colors: desaturated colors are less regulated between organs, whereas more saturated colors are differentially regulated. Genes that are ubiquitous and stably expressed are desaturated blue (e.g., ALG2/6), whereas the genes with a large IQR (or spread) in expression values between fewer specific cells is a bold red color (e.g., B3GNT6). (B) Illustrative examples of histograms of normalized gene expression values for ALG genes that have narrower spreads (top) and for sialyltransferases that have broader spreads for expression values (bottom). Histograms for all glycogenes are available in Figure S3A. Glycan symbols are drawn according to the SNFG format (Neelamegham et al., 2019). See also Figures S2, S3 and S6.

We chose to use a minimum cut-off of 1 CPM (as used by the GlycoMaple tool (Huang et al., 2021)) for the normalized organ RNA-seq data, and calculated the overall repertoire of glycogenes with CPMs (as calculated before normalization to housekeeping genes) that pass this cut-off for each organ, plotting the percentage of organs where each glycogene CPM passes the cut-off filter (Figure 2A, full figure in Figure S2A). The data revealed that most glycogenes are minimally expressed in at least 66% of organs, and a minority are expressed specifically in fewer than 33% of organs. The genes expressed in fewer organs (orange/red color) are likely specifically expressed in these organs, and so are to be considered hotspots in regulation of glycosylation at the organ level.

Next, we chose to quantify variability in transcript quantitation across all organs by calculating the IQR (spread) of CPM values for each glycogene for all samples contributing to the organ data. To avoid issues with low expression of glycogenes, we again apply the 1 CPM cut-off before applying housekeeping gene normalization, and then calculating the IQR values. We expect that the glycogenes that follow the same transcriptional programming in different organs (i.e., the same quantity of transcripts for the gene are present in different organs) will correspondingly have low IQRs, whereas those with non-uniform expression between organs (i.e., there are different quantities of transcript for the gene in different organs) will have higher IQRs.

For most glycogenes that we examined, the IQR of CPM values varied from 0.0–0.9 (approximately a 2.5-fold change from lowest to highest), this is a narrow range, best exemplified with the ALG family of genes (Figure 2B, top histograms). In contrast to glycogenes with narrow spreads, a subset of genes have IQRs greater than 0.9, (greater than an approximate 2.5-fold between lowest and highest expression), exemplified by the ST3GALs Figure 2B, bottom histograms). The 33 genes with the largest difference in expression between organs have at least a 4-fold difference between the lowest and highest expression level. The genes with the largest IQRs are candidates to be considered as hotspots in regulation of glycosylation. Full histograms for all glycogenes are available in Figure S3A.

We visualized the IQR data in combination with the information collected about overall capacity per organ, which revealed additional hotspots of regulation on the heatmap (Figure 2A) in addition to the red/orange hotspots. These hotspots are associated with genes that are both ubiquitously expressed and regulated to different levels of expression either within or between organs (fully saturated blue color). Between the hotspots related to overall capacity, and those related to IQR, the hotspot glycogenes are largely located in the elongation and capping steps, or are members of large isoenzyme families, in agreement with our previous observations when calculating variability using Tau (Joshi et al., 2018).

### Patterns of regulation at the single cell level

#### Sourcing of single cell transcriptomics data

We next aimed to investigate how to use single cell data to reveal whether differences in glycosylation capacity and variability in expression levels are also seen between single cells in organs. We chose to use a repository of uniformly processed single cell data (The Tabula Sapiens Consortium, 2022) because of the quality of cell type annotations made available for each cell as part of the dataset, and the confidence in the uniform quality of data coming from a single consortium. The Tabula Sapiens data is a first draft single cell transcriptomic atlas comprising data from 24 different tissues and organs and a total of 162 unique cell types are annotated in this dataset.

Although we based most of our analysis on human data, we also used mouse single cell datasets (from PanglaoDB (Franzén et al., 2019)) to validate select observed patterns and to increase the coverage of neuronal cell types. In summary, from both the data sources we selected data produced using Chromium 10X with unique molecular identifiers (UMI) and smartseq2, covering approximately 24 organs in human and 30 organs in mouse (Figure 1C).

#### Calculating glycogene expression levels for individual cell types

Single cell RNA-seq analyses typically take advantage of dimensionality reduction methods followed by subsequent clustering of datapoints for single cells (Butler et al., 2018) to identify which cells have similar transcript profiles and thus can be inferred to belong to a cell type by interrogating marker genes. For the data we selected to analyze, we relied on both the identified clusters of single cells and the cell type annotations as calculated by the maintainers of Tabula Sapiens and PanglaoDB. Clusters of single cells can generally be considered representative of a particular cell type, and hence referred to as one cell type. The clustering process determines a boundary between cell types, and does not adequately capture intermediate states in processes such as maturation, or include more dynamic aspects such as the state of the cell cycle. Because the data collected for each single cell in a cluster carries high error rates and are prone to technical dropouts, we had to use clusters of single cells instead, which provides an *in-silico* amplification of the gene expression data by combining counts for a cluster (i.e., an enrichment of cells of the same cell type). We treat these clusters as pseudo-bulks for that individual cell type, and the corresponding mRNA gene expression quantitation from the cluster as a pseudo-bulk transcript quantitation (Lun et al., 2016), referred to as a pseudo-bulk quantitation from here on (see STAR Methods for calculation). After filtering and basic quality control, we calculated the pseudo-bulk quantitation for all genes for 229 individual cell types from Tabula Sapiens (Human), and 2317 individual cell types from PanglaoDB (Mouse).

Apart from the previously mentioned limitations regarding the quantitation of lowly expressed genes and technical dropouts, the analysis of single cell data must consider factors that can affect downstream analysis. A major factor is that grouping cells into clusters is highly dependent on the choice of algorithm and the parameters chosen when applying the algorithm, such that any group of cells could be decomposed into smaller clusters of cells given the right application of a clustering algorithm. Similarly, larger clusters may be decomposed into smaller clusters even though the cells that comprise the cluster may be similar in identity. Clusters of single cells that are made up from larger numbers of cells are more reliable for calculating the pseudo-bulk quantitation for a gene, especially when the gene is expected to be expressed lowly. Rarer cell types, that are assigned only to smaller clusters (<200 cells in a cluster, and approximately half of all cell types from the datasets) will likely be overlooked by our methodology.

#### Pseudo-bulk quantitation values from single cell data are broadly similar to organ-level expression data

To determine if pseudo-bulk quantitation values for all individual cell types reflect the overall levels and variability of expression from organ data, we normalized the pseudo-bulk quantitation values for each gene for each individual cell type using the same set of housekeeping genes used in the organ-level analysis. Next, we log-transformed these values, and calculated the IQR and mean. For most of the glycogenes, these normalized pseudo-bulk quantitation values showed approximate agreement with the organ level transcriptomic data (Figure S3A). Overall, the means of the normalized transcript quantitation values (i.e., CPM or pseudo-bulk quantitation) for each gene correlated reasonably well between organ and single cell data (Pearson correlation coefficient, r = 0.63, two-tailed t-test, p = 2.68E-26, n = 222, Figure S3B), whereas the correlation of IQRs showed poor global correlation (Pearson correlation coefficient, r = 0.46, two-tailed t-test, p = 8.03E-13, n = 222, Figure S3C).

We noticed that in general, the IQR was higher for normalized pseudo-bulk quantitation values compared to the corresponding values from organ data, which we thought could be explained by the increased granularity of single cell data reflecting more variation in gene expression levels than the average values from heterogeneous cell type populations in organ data, and abundance data for each cell type being erased during the transformation into cell type level data. We plotted the correlation of IQR between organ and individual cell type data, binning correlations by organ-level IQR and finding that the highest correlation could be seen for organ-level IQRs in the interval between 0.5 and 1 (Pearson correlation coefficient, r = 0.43, two-tailed t-test, p = 2.71E-06, n = 108, Figure S3D), which confirmed that the correlation between IQR at the organ level and individual cell type level is strongest for lower IQR values. At higher IQR values, both the strength of correlation, and significance drop, lowering overall confidence in the correlation. Together this indicates that expected IQRs at the cell type level can most reliably be estimated from organ level data for glycogenes with lower IQRs.

To further verify that we were estimating the ranges of values for transcript correctly, we used an alternative method to estimate the real expression values from underlying single cell data. We selected scTransform (Hafemeister and Satija, 2019) to calculate gene-wise expected expression values. For a small set of available benchmark data, we applied this method to count data across all cells within a dataset to estimate the ranges of expression values and validate our calculation method for pseudo-bulk quantitation values. After performing the same set of normalizations and log-transformations as for the organ RNA-seq and pseudo-bulk quantitation data, a visual inspection of the data showed that the ranges of values were similar, confirming that our transformation of single cell data into cell type level, pseudo-bulk quantitation data did not introduce any large discrepancies into the data (Figure S3A).

The reasonable agreement between the organ and pseudo-bulk quantitation estimates of gene expression implies that we could make use of organ-level data to estimate upper and lower expression values for each glycogene, assuming that the ranges we derive from the organ-level data is representative of the typical range of expression of glycogenes in healthy humans, as all samples from the organ level data are from healthy donors. Normalized pseudo-bulk quantitation values that lie between the upper and lower values, indicate that the gene is expressed in the typical healthy range, whereas normalized quants outside the range of healthy upper and lower expression values are correspondingly highly and lowly expressed. We collated this reference range data into a reference table (Table S3), and incorporated this into an R package, so that we could predict whether a glycogene is not expressed, lowly expressed, or highly expressed – based only on the data present in a user-supplied single sample or cluster from a single cell analysis.

Although most glycogenes demonstrate a general agreement between the ranges of values for bulk and single cell data, for a minority of genes the ranges of values are offset with respect to the bulk values (both higher and lower) (Figure S3A). This error is particularly prominent for e.g., the *Galnt2* gene in the mouse data, where very few transcripts are detected for this gene, despite substantial evidence that it is in fact expressed.

### Metrics to identify hotspots of regulation for single cells

#### Overall glycosylation capacity in individual cell types

As with organ-level data, we next calculated which individual cell types have minimal expression of each glycosyltransferase so as to estimate the overall glycosylation capacity. To realize this, we calculated a cut-off for pseudo-bulk quantitation values that would work similarly to the 1 CPM cut-off we used for organ data. Using a human HEK293 cell line reference dataset where both bulk RNA-seq and single cell RNA-seq was performed (Ding et al., 2020), we examined the relationship between the bulk RNA-seq TPM values and calculated pseudo-bulk quantiations. The highest pseudo-bulk quantitation values for all genes ranged up to approximately 4.5, whereas TPM values ranged up to approximately 10,000 (Figure S4A). Glycogenes in HEK293 cells are expressed up to a pseudo-bulk quantitation value of approximately 1, and bulk RNA-seq TPM of 100 (Figure S4B), and the TPM and pseudo-bulk quantitations showed good agreement with each other (Spearman correlation coefficient, r_s_ = 0.825, one-tailed t-test, p = 3.1E-45, n = 177, using all cells). Given the good agreement, we proceeded with estimating the cut-off as a minimal pseudo-bulk quantitation (Figure S4C), and margin of error as a function of the number of single cells that make up the cluster for the pseudo-bulk. In this way, we could calculate the confidence that pseudo-bulk quantitation values are above or below the minimal cut-off, with more certainty given to pseudo-bulk quantitation values calculated from clusters containing more cells due to smaller margins of error.

**Figure 3:**
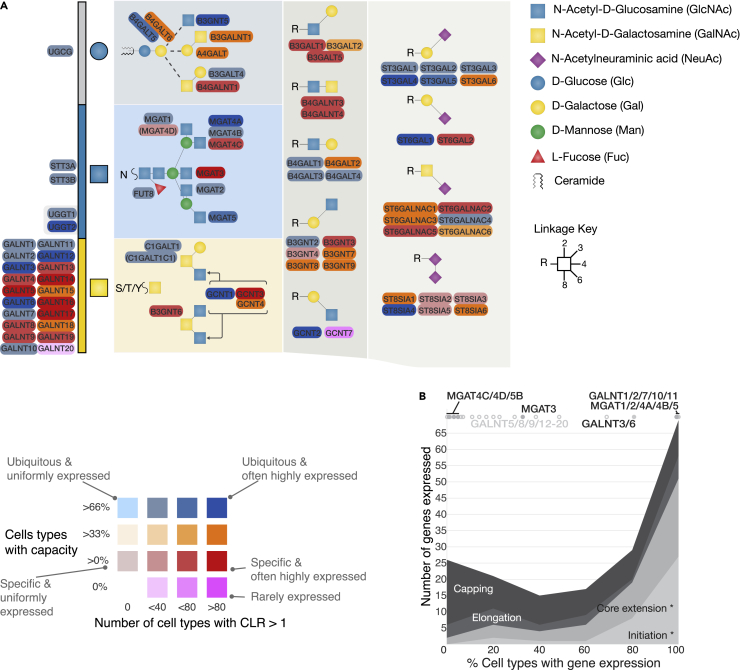
Identification of hotspots of regulation in the glycosylation network from single cell data (A) As with Figure 2, an extract of the glycogene based heatmap indicating hotspots of regulation. The full heatmap is available in Figure S2B. An increased number of hotspots are visible on the heatmap when the granularity of the data is increased. The overall glycosylation capacity, as percentages of cells that genes are expressed in (from none to over 60% of cell types) are plotted using color on a heatmap, whereas an indicator of specific expression - the number of cell types that that have higher expression of each gene (relative to all cells) modulates color saturation. Genes that are expressed more highly in more cells use saturated colors. (B) The percentage of cell types that express glycogenes from the different gene categories (See rainbow figure, Figure S1). The initiation and elongation genes in the GALNT and MGAT isoenzyme families do not contribute to shape areas and are shown at the top of the plot because of their behavior being significantly different from the other genes in their respective groupings. Glycan symbols are drawn according to the SNFG format (Neelamegham et al., 2019). See also Figures S2, S4, S5, and S6 and Table S4.

Similarly to the organ level analysis, we defined overall glycosylation capacity based on the set of glycogenes whose pseudo-bulk quantitation values for an individual cell type passed the cut-off filter (Figure S5A). The percentage of individual cell types that expressed each gene above the cut-off value was plotted in a heatmap (excerpt in Figure 3A, full figure in S2B). The data indicates that the capacity to initiate glycosylation across all glycosylation pathways is present in nearly all cell types identified from the reference dataset (major tissues, and the total number of cell types from each tissue that we examined are listed in Table S2). However, in contrast to the organ-level data, many of the glycogenes identified as ubiquitously expressed at the organ level are only expressed in up to 33% of cell types, indicating as expected that more differential expression of other glycogenes are “hidden” in the heterogeneous cell source from organ data. Despite this we observed that approximately 60% of the glycogenes are expressed in at least 50% of cell types, and 50% are expressed in at least 80% of cell types (Figure S5B). This suggests that a large proportion of the glycogenes are not regulated by being switched on and off between cell types.

#### Higher than average expression as a hotspot of regulation for an individual cell type

Single cell data offers the opportunity to identify the cell types where glycogenes are heavily regulated. Instead of using the IQR across all cell types, we looked to find the individual cell types where glycogenes were more highly expressed compared to the average level in all cells. We chose not to investigate lower expression of genes because these lower bounds of expression likely overlap with the limits of detection for single cell RNA-seq. The centred-log-transform (CLR) of pseudo-bulk quantitation values can quantify the magnitude of variation from the mean values across all cell types. We calculated the CLR for all glycogenes in all cell types and integrated this measurement into the heatmap (excerpt in Figure 3A, full figure in S2B). The CLR enabled us to not only identify the hotspots of regulation in a cell-type manner, but also to find the genes that demonstrate little variation of expression in most cell types. 63 genes were expressed highly (CLR value >1) in between 45 and 77 individual cell types (out of 229 total), 58 genes are expressed highly in between 22 and 44 of cell types, and 102 genes were rarely highly expressed (at most 21 individual cell types), including many members of the ALG genes, STT3A/B, MGAT1 and MGAT2, GALNT2 and POFUT1/2. The gene that is most commonly expressed at higher levels amongst cell types is MFNG (Figure S2B). The data reflects the trends observed from the organ data, where elongation and capping genes as well as isoenzyme families are more regulated than initiation and core extension genes (Figure 3B).

### Overall glycosylation capacities of a single cell, and hotspots of the glycosylation network

The combined overall glycosylation and hotspot data (Figure 3A) reveals additional detail over the simplistic model of binary regulation of capacities between cell types. The data highlights that amongst the elongation and capping steps, certain features are equally as ubiquitous as those in initiation steps (e.g., type2 N-acetyl-lactosamine (LacNAc) chains and α2-3 sialylation). This ubiquity is provided for by “workhorse” genes in isoenzyme families that vary little in expression and are always present in most cell types (e.g., B4GALT1/3/4, B3GNT2 and ST3GAL3). Other isoenzyme families have more members that are regulated by toggling expression in individual cell types, supplementing the capacity of workhorse genes. Finally, a minority of genes can be ubiquitously expressed but also are hotspots of regulation, showing capacity for modulation (as evidenced by higher CLR values), boosting expression in a subset of cell types (as seen in for example, the sialyltransferases).

Although the initiation steps appear rather ubiquitously available in cells, it is important to note that the initiation of the GalNAc-type O-glycosylation orchestrated by the large family of the polypeptide GalNAc-transferases (GALNTs) has members that exemplify each of these patterns of regulation: Five workhorse enzymes (GALNT1, GALNT2, GALNT7, GALNT10 and GALNT11) are ubiquitously expressed and are mostly not regulated between cell types in Tabula sapiens (light blue); whereas GALNT3/6/12 are hotspots, often up-regulated in different cell types (over 80 cell types). The remaining 12 GALNTs are most regulated and expressed in specific cell types (red).

The capacity to perform sialylation is ubiquitous, and all cell types have the capacity to catalyze sialic acid linkages: α2-3 (mostly provided by ST3GAL1-5), α2-6 Gal (mostly provided by ST6GAL1), α2-6 GalNAc (mostly provided by ST6GALNAC4), and α2-8 linkages (mostly provided by ST8SIA4). The expression patterns of the ST3GALs are unique with respect to this family, as our understanding of the specificities of these transferases implies that at least for O-linked (ST3GAL1/2) and glycolipid (ST3GAL3/5) sialylation, these genes are co-expressed ubiquitously as seemingly redundant pairs. All cell types are predicted to have capacity for α2-3 sialylation of O-glycans, 27 cell types are predicted to lack α2-3 sialylation capacity for N-linked glycans, although for 22 cell types this activity could possibly be provided for by ST3GAL3. On top of a common capacity for sialylation, this capacity may be “boosted” (i.e., the total expression of enzymes from within an isoenzyme family increases) through higher expression of the hotspot genes ST3GAL4/5 in at least approximately 20% of cell types.

### Three distinct patterns of glycogene regulation identified

**Figure 4:**
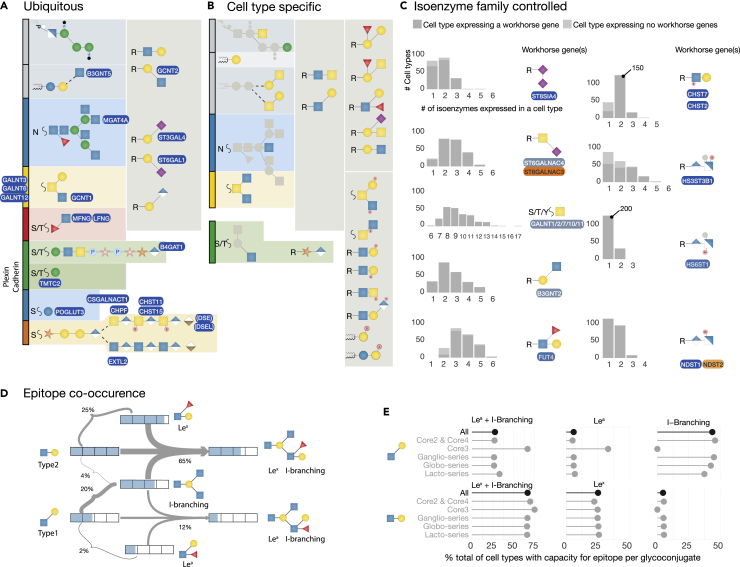
Common and unique glycosylation capacities of cell types (A–C) The three main patterns of hotspots of regulation for the glycosylation network, summarizing data from Figures 3A and S2B. The ubiquitously expressed workhorse glycogenes label the glycosylation feature synthesised by them. Workhorse genes labels are shown with a background fill matching the colors used in the heatmap from Figure 3A. (A) A core glycosylation capacity is common to all cell types, synthesized by glycogenes that are ubiquitously expressed, mostly showing little regulation between cell types, with the exception of the features labeled by genes in bold, that could be regulated from cell to cell. Not shown are the ubiquitous pathways for EOGT, Core M1 O-Mannosylation, C-Mannosylation, POGLUT1/2 type and POFUT2 type EGF glycosylation, collagen glycosylation and OGT nucleocytoplasmic glycosylation. (B) Celltype specific glycan features that are synthesised by glycogenes that are specifically regulated to cell types. (C) Glycan features that are encoded for by glycogenes that are members of isoenzyme families, of which one member is ubiquitously expressed, but the other members demonstrate patterns of cell type specificity. The bar charts show the number of cells that a single member up to all members of the isoenzyme family is expressed in. The dark portions of the bar charts indicate when at least one of the workhorse genes is expressed, whereas the light gray portion indicates the number of cells where none of the workhorse genes are expressed. (D) Estimation of the uniqueness of glycan epitopes that can be encoded on PolyLacNAc chains. The most unique cells (approximately nine cell types for each) can produce Le^a^ structures without any I-branching present or produce type2 chains without any Le^x^ present at all. (E) Uniqueness of PolyLacNAc features (I-branching, Le^a/x^) broken down by cells that can produce specific glycoconjugates. Glycan symbols are drawn according to the SNFG format (Neelamegham et al., 2019).

Figure 4 summarizes the predictions for glycosylation deducible from our analysis and presents the key findings of cell type specific regulation of glycosyltransferases. Three general themes in regulation of the glycosylation network emerge from our analysis:

1. Ubiquitous glycosylation. Nearly all cell types are predicted to express the glycogenes required to initiate glycosylation in each of the 17 major strict pathways, and to a large extent are also predicted to have the capacity to perform core extension in these pathways (Figure 4A). Glycogenes that follow this pattern of regulation are all at least minimally expressed and pseudo-bulk quantitation values demonstrate little variability between cell types (low CLR values). Where the capacity is encoded for by an isoenzyme family, these are all/mostly ubiquitously expressed, leaving little possibility for binary cell type regulation at the transcriptional level. Where expression is most variable between cells (e.g., B3GNT5), these specific genes show evidence that they could be often modulated at the mRNA level between cell types (high CLR values).
2. Cell type specific glycosylation. A group of glycosylation features are encoded for by genes that are primarily expressed in a cell type specific manner (Figure 4B). These features include the blood group antigens (ABH, Lewis, SE), most non-GAG sulfation (including sulfatide biosynthesis), LacDiNAc and type1 chain LacNAc extensions, as well as the polymerization steps of matriglycan extension (i.e., the extension by LARGE1/2). Also cell type regulated are GPI side chains, ganglio and globo-series glycolipid biosynthesis (Fenderson et al., 1987; Kannagi et al., 1983; Liang et al., 2010), bisecting GlcNAc (known to be tissue-specific (Miwa et al., 2012)) and Core3/4 O-GalNAc (Iwai et al., 2005) and Core M2 O-Man glycans (Sheikh et al., 2017).
3. Glycosylation by members of isoenzyme families that include workhorse glycogenes. Glycosylation steps directed by families of isoenzymes with partial redundant functions pose challenges in predicting outcomes of their expression. Because the individual specificities of members of the isoenzyme family often remain obscure, it is hard to predict whether co-expression of members of isoenzyme families is, for example, a cellular response to an increased workload for this particular type of glycosylation, or whether distinct activities of the isoenzymes are required. The patterns of expression of glycogenes within this group (Figure 4C) suggest that the latter explanation is favored. Each isoenzyme family includes one or more workhorse glycogenes that provide for glycosylation capacity in nearly all cell types. However, most cell types will typically express between 2-3 members of the isoenzyme family (although the GALNTs are expressed together typically in groups of 8–9), and the remaining isoenzymes are typically sporadically expressed in different cell types.

### Transcriptional regulation of glycogenes cannot encode for unique epitopes

Glycan recognition by receptors (e.g., antibodies (Temme et al., 2021), selectins (McEver, 2015), lectins (Bojar et al., 2021), and carbohydrate-binding modules (Lombard et al., 2014)) mediates selective recognition of cells. The recognized features are generally well-defined with respect to terminal glycan structures as presented in Figure 4D, whereas it appears that recognition for at least some glycan-binding proteins involve more complex features (Cohen and Varki, 2014). Nevertheless, we attempted to estimate the minimum number of cells that can be addressed by programming glycosylation to present unique terminal glycan epitopes. Around 20 glycans features show evidence of restricted expression to specific cell types (Figure 4D). Type1/2 chain biosynthesis and elaboration provide for an opportunity to synthesise unique epitopes that can be read by molecules such as galectins and selectins. We calculated the number of cells that have capacity to produce either type1 or type2 chains, Large-I branching and Le^x/a^ fucosylation, to estimate the number of cells that can produce unique epitopes based on these features (Figure 4D). The most unique cells are those that can produce Le^a^ without branching, or branched type2 chains without fucosylation. The least unique cells are those that can produce branched type2 chains with Le^x^, followed by Le^x^ without branching. Across glycosylation pathways (Figure 4E), cells that can produce Core3 glycans demonstrate the most specific programming for LacNAc chain extension, with the capacity to synthesize branched LacNAc chains and Le^a^ in most cells. Cells that produce lacto-series glycolipids also demonstrate a slight specificity toward synthesis of Le^a^ and I-branching together.

### Examples of hotspots in the glycosylation network correlated with experimental results

**Figure 5:**
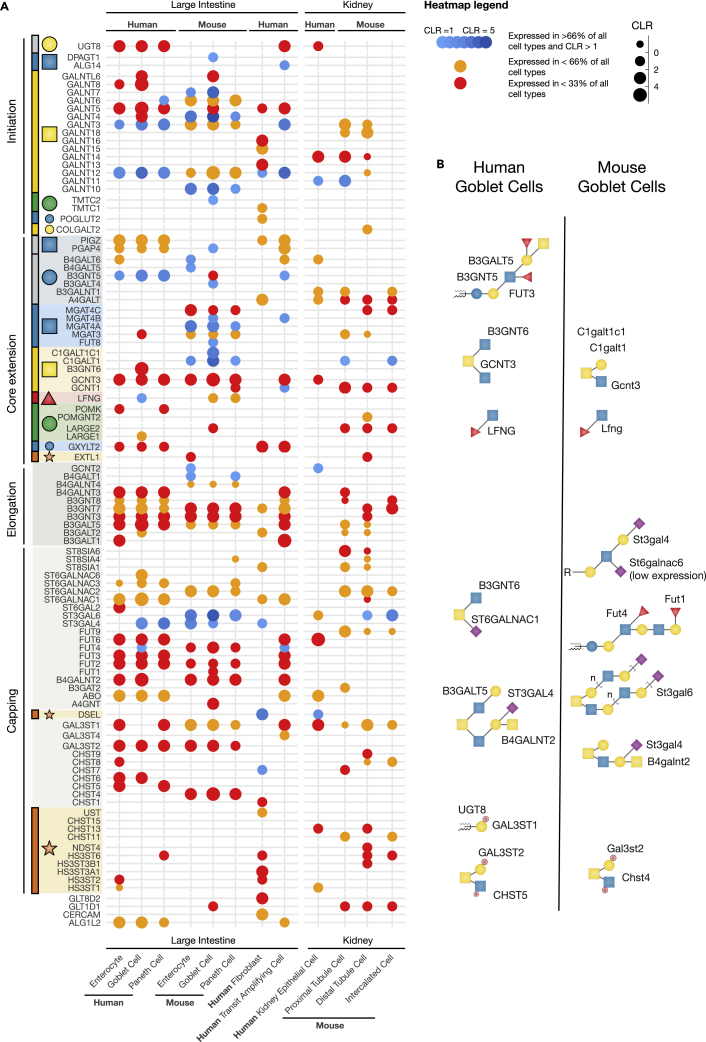
Predicted hotspots of regulation from single cell analyses of multiple organs (A) Heatmap of the predicted hotspots of regulation on the glycosylation network for cells from human large intestines and kidneys, as well as mouse large intestine and kidneys. The predicted regulated genes are arranged according to the rainbow figure, into initiation, core extension, elongation and capping groups. Pathway specificity is indicated by the initiating monosaccharide for the pathway. Heatmap points are colored according to the criteria used to include the gene as a hotspot: If the gene is ubiquitously expressed, the CLR is used to give a blue of varying saturation (most saturated is the highest expression), and if the gene is specifically expressed it is colored orange or red if it has been observed expressed in fewer than 66% or 33% of cells respectively. The CLR for genes scales point size. (B) Example glycan structures predicted to be synthesized in goblet cells in mouse and human, based on the regulated glycosylation capacity are drawn. The glycans are labeled with the genes that regulate the feature from the hotspot heatmap. Glycan symbols are drawn according to the SNFG format (Neelamegham et al., 2019). See also Table S3.

We next delineated hotspots of regulation in different cell types of two organs (large intestine and kidney), from human and mouse (Figure 5A). Lymphocyte and unknown cell types were filtered from the large intestine and kidney datasets, and cell types from clusters that included fewer than 200 cells were not included, which reduced the number of cell types available for each organ to those presented in Figure 5. The human dataset from the large intestine includes enterocytes, goblet cells, Paneth cells, fibroblasts and transit amplifying cells. The mouse data includes subdivisions for enterocytes, goblet cells and paneth cells. The kidney cells are annotated as “epithelial cells” from human, and given their expression of LRP2, we can assume they are functional differentiated proximal tubule cells. Mouse kidney cells include proximal tubule cells, distal tubule cells and intercalated cells. Mouse kidney cell types also include incorrectly annotated “acinar cells” which were annotated with a much lower confidence than the majority of acinar cells annotated in PanglaoDB. We excluded these cells from the heatmap.

Extracting the hotspots of regulation from our analysis, we identified a set of 99 genes as hotspots of regulation across all the cell types. The patterns of predicted hotspots of regulation demonstrate organ and species similarities, e.g., intestinal cell types from human are often regulated more similarly with each other, than between mouse and human. Among the identified hotspots we were able to correlate several of these with experimentally determined glycosylation changes. For example, the GALNT11 isoform is known to be widely expressed in cells and with a remarkably high level of expression in the kidney proximal tubule epithelial cells (Schwientek et al., 2002), where the specific role of GALNT11 has been described (Tian et al., 2019; Wang et al., 2018), and although the gene is ubiquitously expressed, it is accordingly seen with higher expression in kidney proximal tubule epithelial cells (Figure 5A).

An interesting species (mouse-human) difference in O-glycan core structures has been described for goblet cells, with both common and unique differences in GalNAc-type O-glycosylation capacity between these cells in different organisms. We investigated these changes in detail using the heatmap (Figure 5A), illustrating the different and common capacities for glycosylation (Figure 5B).

#### Initiation/core extension

47 glycogenes involved in pathway specific initiation and immediate extension steps of glycosylation have been identified as hotspots of regulation. GALNT4/Galnt4 are predicted to be expressed in goblet cells, and querying the data reveals that MUC1/Muc1 is also expressed in goblet cells. This correlates with the role of GALNT4 in glycosylating the single threonine glycosite (PDTR motif) in the tandem repeat sequence of MUC1, as originally determined by *in vitro* assays (Hassan et al., 2000) and more recently also shown in HEK293 cells (Nason et al., 2021). The GalNAc-type O-glycosylation pathways split at the core extension/branching step between a pathway to synthesize Core1 and Core2 glycans, and the pathway for synthesis of the Core3/4 glycans. Both species have capacity for synthesis of Core1 and Core2 glycans (C1GALT1/C1GALT1C1 and GCNT3 respectively in humans), whereas only humans express B3GNT6 in goblet cells, and so the capacity to produce Core3 and Core4 glycans is not predicted to be present in mouse (Arike et al., 2017; Thomsson et al., 2012).

Goblet cells in human also demonstrate a boost in expression of LFNG, which appears to be in agreement with a role of this modification in maintaining intestinal homeostasis (Kadur Lakshminarasimha Murthy et al., 2018). The high expression of B3GNT5 in humans supports previous reports of lacto-series glycolipid biosynthesis in the intestine (Holmes et al., 1987; Magalhães et al., 2015). The high expression of the ganglio-series transferase B3galt4 is in agreement with mass spectrometry data for mouse intestines (Arike et al., 2017).

#### Elongation/capping

Human goblet cells produce primarily type1 LacNAc chains (human specific B3GALT5), whereas mice can produce repeated LacNAc chains using the default type2 chain pathway (similarly to humans (Holmes et al., 1987)). Furthermore, these chains are commonly fucosylated by FUT4 between the two organisms, whereas humans also express FUT6 and FUT3 (the latter of which can synthesize type1 Lewis, or Le^a^ structures). As suggested by the transcriptomic analysis, Core2 O-GalNAc structures are likely sulfated in mice (Arike et al., 2017). Mice perform internal α2-6 sialylation on the GlcNAc residue of LacNAc chains (Thomsson et al., 2012), and ST6GALNAC5/6 have been reported to be able to synthesize disialyl Le^a^ glycans (Tsuchida et al., 2003). Our analysis supports evidence for low expression of St6galnac6 in goblet and Paneth cells (data in Table S3), alongside the expression of B3galt5 to synthesize type1 LacNAc chains (Figure 5A). The expression of ST6GALNAC6 is higher in human goblet cells compared to mice. In contrast to this, α2-6 sialylation on GalNAc is exclusive to humans, and is likely catalyzed by ST6GALNAC1, whose expression is supported by the transcriptomics. Despite the predicted expression of ST6GAL2 in goblet cells, there is little evidence for α2-6 Gal sialylation, as SNA does not bind in colon, even after unmasking with a deacetylase (Murayama et al., 1997), and other data suggests that ST6GAL2 has very limited specificity (Krzewinski-Recchi et al., 2003; Takashima et al., 2003).

Beyond the intestinal and kidney data presented in the heatmap, we also uncovered evidence that corroborates a unique expression pattern for B3gnt6 in the mouse, where we predict that many neuronal cell types express this gene (data in Table S3). This was unexpected based on our understanding of the limited expression of Core3 glycans in mucosa. RNA-seq data from Human Protein atlas and GTeX supported quantification of B3gnt6 transcripts in the brain. Tabula sapiens included very little human neuronal data, but a recent report on glycomics of the human brain reported that Core3 glycans could be detected (Wilkinson et al., 2021).

The well-characterized HNK1 epitope is primarily regulated by the B3GAT1 enzyme, and has been described as being primarily expressed in neuronal tissues. The human dataset we are analyzing has few neuronal cell types included, yet we predicted that a number of cells had the capacity to produce this epitope. We predict expression of B3GAT1 in a small group of cell types that correlates with experimental detection of expression of CD57: T-cells (Markey and MacDonald, 1989); photoreceptor cells and Müller cells (Uusitalo et al., 2003); and epithelial cells from the prostate (Wahab and Wright, 1985) (data in Table S3).

## Discussion

We posit that to accommodate the diverse and distinct glycosylation pathways co-occurring in the ER and Golgi compartments without detrimental crosstalk and interference, the many glycosyltransferases and sulfotransferases involved in glycosylation largely autonomously coordinate the ordered assembly of the glycome using intrinsic sufficient substrate specificities and kinetic properties. Here, we have taken a first step toward probing the regulation of the glycome at the individual cell level by analyzing single cell transcriptome data, and although we demonstrate that this is a promising avenue to bypass analytical roadblocks, we also identify the sensitivity and quality of transcriptomics data as the major challenge for success. Cells in complex organisms must regulate glycosylation processes with a high degree of precision to tailor the glycome to fulfill the great variety of known functions assigned (Schjoldager et al., 2020), and decades of immunohistochemical studies with individual cell resolution (Dabelsteen et al., 1991) and more recently MALDI-TOF imaging of N-glycans on formalin-fixed paraffin-embedded tissue sections (currently unable to resolve single cells) (McDowell et al., 2021; Powers et al., 2013), confirm distinct features of the glycome on individual cells and related populations of cells. Transcriptional and/or translational regulation of the enzymes directly involved in synthesis is arguably the easiest way to provide for regulation of the glycome (Huang et al., 2021; Mandel et al., 1990; Paulson and Colley, 1989). Our analyses find that one-third of the glycosylation genes appear to be under little cell and tissue specific regulation, and these genes are characterized by encoding enzymes primarily directing basic initiation and core scaffold assembly of glycans on proteins and lipids (Figure 4). The remaining majority of genes exhibit varying degrees of cell specific regulation in terms of both on-off and high-low expression in different cell types. Many of these could be identified as hotspots in regulation of glycosylation, and as examples, we identified 65 and 109 glycogenes as hotspots of regulation in kidney and intestinal cells respectively, from both mouse and human data. Thus, our study indicates that the part of the core glycosylation machinery that synthesizes the core of the glycome is ubiquitously present in all cells, whereas structural variations including sites of O-glycan attachment and terminal modifications are differentially regulated in cells.

Our analysis of single cell transcriptome data provides a first draft of the transcriptional landscape of the glycosylation genes and predicted glycosylation capacity in cell types. Given limitations in the technology used to gather this data, we could only sketch the broad outlines of the mRNA regulatory landscape and identify apparent hotspots of regulation. The two overriding limitations for our analysis were that (1) the lower levels of expression of glycogenes challenge our ability to quantify expression levels at the single cell level, and thus identify biologically relevant changes in expression; and (2) pseudo-bulk quantitation values are representative of the average expression for a cell type corresponding to a cluster of single cells and as a result is highly sensitive to how clusters of cells are classified. Thus, to fully appreciate the magnitude of transcriptional regulation of glycogenes more accurate quantification of mRNA at the lower end is needed. Nevertheless, we believe that the strategies set out in this study are widely applicable to the rapidly emerging single-cell transcriptomics data (as recently exemplified in a study of pancreatic ductal adenocarcinoma (Rodriguez et al., 2022)), and we aim to apply this to single cell data from all human tissues to reveal the complete diversity in regulation of glycogenes.

### Relation between transcriptional regulation of glycogenes, expression of enzyme proteins, and the glycosylation output

Application of transcriptomics or proteomics provides insights into the expression of the enzymes involved in glycosylation, and thus the components of the machinery that are available to perform glycosylation. This information still leaves a need for transformation of enzyme data to the actual produced cellular glycome. On-off regulation of glycogenes clearly can result in changes in the glycome, a feature that has been exploited in the many knockout studies of glycogenes in animal models (Lowe and Marth, 2003; Stanley, 2016) and more recently in cell lines (Narimatsu et al., 2019). Direct evidence for specific regulation of the glycome by expression of a specific glycosyltransferase is however limited. Investigators have used *in situ* hybridization to probe glycogene expression (Ten Hagen et al., 2003; Rausch et al., 2015; Yamamoto et al., 2004), but direct relationship with expression of the corresponding enzyme and glycan products are generally unexplored. To our knowledge one of the few examples of direct analysis of enzyme protein and corresponding glycan product is the co-appearance of the blood group A glycan and the blood group A glycosyltransferase in cells during epithelial differentiation in stratified squamous epithelia (Mandel et al., 1990). The stratified squamous epithelium with its well-defined cellular maturation and differentiation pathway provided some of the earliest evidence for tight regulation of the cellular glycome in stepwise fashion as cells move upwards toward terminal differentiation (Dabelsteen et al., 1984; Mandel et al., 1991), and the characteristic loss of blood group A in oral cancer was also accompanied by loss of the glycosyltransferase (Mandel et al., 1992). However, the latter study also highlighted that expression of the blood group A glycosyltransferase is necessary, but not sufficient for production of the blood group A glycan as found in colon tissue, where the absence can be explained presumably by competitive mechanisms with other glycosyltransferases and lack of substrate. These studies, however, did not include analysis of the transcriptional regulation of the blood group A gene in the cells. A general void in appropriate antibodies to glycosyltransferases has hampered direct correlations of enzyme protein expression with gene transcript and glycan products at the individual cell level (Steentoft et al., 2019). Furthermore, few examples of transcriptional regulation of glycosyltransferases in direct response to specific functional requirements exist, and perhaps the only with direct experimental support is the transcriptional regulation of GALNT3 in response to phosphate to serve its role in regulating FGF23 and phosphate homeostasis (Chefetz et al., 2009; Takashi et al., 2019).

The consequences of graded low-high expression of glycogenes on the glycosylation output are unclear. Whereas it seems logical that an increase in the amount of a glycosyltransferase enzyme in a cell would lead to an increase in glycosylation efficiency, this may not be the case given the complex and intertwined pathways of glycan assembly, where multiple enzymes utilize the same substrates, and isoenzymes have overlapping functions but potentially different subcellular localizations. It is conceivable that most glycosyltransferases in fact are present at supersaturated levels given that our transcriptional analysis suggest rather ubiquitous expression levels and, for example, that extremely high levels of expression of recombinant therapeutic glycoproteins in mammalian cells do not appear to exhaust the cellular glycosylation capacity (Narimatsu et al., 2021). We previously explored the effect of graded expression of two polypeptide GalNAc-transferases (GALNT2 and GALNT11) (Hintze et al., 2018), and interestingly found that enzyme expression levels did not affect their redundant contributions to the O-glycoproteome presumably because GalNAc-transferase isoenzymes for these substrates are already supersaturated, whereas their non-redundant contributions for a few specific protein substrates were tightly regulated by the enzyme dose. This is likely applicable to all the many glycosylation steps that are regulated by isoenzymes with partly redundant functions, and installed to enable differential regulation of select features of the glycome.

### Transcriptional and translational regulation of glycosyltransferases

Limited knowledge of mechanisms of transcriptional regulation of most glycogenes is available, although considerable information has been accumulated for select genes such as B4GALT1, ST6GAL1, and OGT. Variation in splicing of glycogenes does not appear to be a major factor for glycosylation enzymes, as functional variants have only been identified and characterized for a few genes. The relatively simple domain structures of most glycosyltransferases (for GT-A and GT-B, a single domain) means that these genes are less amenable to regulation of catalytic activity through alternative splicing. However, enzymes with a multi-domain architecture offer more opportunities for splicing to modulate function. For example, OGT encodes for three splice isoforms of the enzyme, which differ in the number of N-terminal tetratricopeptide repeat motifs (Lazarus et al., 2006). GALNT13 can be alternatively spliced, resulting in changes to the lectin domain (Festari et al., 2017), although this is not a major factor for most members of the GALNT family (Bennett et al., 1998). Splice isoforms can also affect the membrane tethering of glycosyltransferases, for example, an alternative isoform of ST6GAL1 without a stem and transmembrane domain exists (Dall’Olio, 2004; Dorsett et al., 2021), or B4GALT1 can encode for both a long and short isoform, the former of which localizes to the cell surface (Lopez et al., 1991). The regulation of glycosyltransferase residency time occurs via control of retrograde trafficking (Liu et al., 2018; Welch et al., 2021) and Golgi dynamics as well as by limited proteolytic processing in the juxtamembrane regions (for type 2 transmembrane GT-A/B fold enzymes) by e.g., SPPL3 and furin (Shifley and Cole, 2008; Voss et al., 2014). The sialyltransferase ST6GAL1 is regulated by BACE-1 cleavage producing a secreted ectodomain (Kitazume et al., 2001; Woodard-Grice et al., 2008) that has been proposed to serve important roles in extracellular glycosylation of immunoglobulins (Irons et al., 2020). The Drosophila fringe glycosyltransferase regulating O-Fuc O-glycan extension on Notch is largely secreted but could be retained functionally with a chimeric GALNT2/fringe design to demonstrate glycosylation function (Brückner et al., 2000). Three human fringes exist, and lunatic fringe (LNFG) is similarly to the single Drosophila fringe mainly secreted through furin proprotein processing (Shifley and Cole, 2008), and the regulation of this cleavage is essential for vertebrate somitogenesis (Shifley et al., 2008; Williams et al., 2016). Finally at the individual protein level, local steric constraints on glycosyltransferases can also affect the final glycosylation found on proteins (Thaysen-Andersen and Packer, 2012).

### Predicting glycosylation capacities and the glycome

Prediction of the glycome so far has largely focused on a few well-studied glycosylation pathways (N-linked and O-GalNAc type) to build mechanistic models of the pathway based on kinetic models of glycosylation (Fisher et al., 2019a,2019b; Krambeck and Betenbaugh, 2005), linear model simplifications that improve the computational tractability of kinetic models (Spahn and Lewis, 2014), or design of capacity based models that combinatorically predict glycan repertoires based on glycosyltransferase reaction patterns (Huang et al., 2021; Kawano et al., 2005; McDonald et al., 2016). Approaches to prediction of the glycome that calculate the glycome output from enzyme kinetics and substrate specificities provide power in their ability to capture the part of the glycosylation process involving coordination of machinery components, but modeling the complete glycosylation process in this manner is a formidable challenge, which is amplified by the dearth of data to support this task. Systems approaches to prediction of the glycome have proven their utility by recapitulating the coordination of glycosylation through intrinsic properties, and working around the limitations of the input data (structural data that is influenced by lab-to-lab variation (Ito et al., 2016), and convoluted measurements of the glycome from heterogeneous cells), but less heterogeneous glycomic profiling data is likely required to recapitulate other factors affecting glycosylation in a computational model. Along these lines, DNA-barcoded lectin methods that link the transcriptome of single cells to the lectins that bind to the cellular glycome provide a possible direction for collection of this data (Kearney et al., 2021; Minoshima et al., 2021). We are optimistic that a combination of existing prediction approaches with machine learning approaches to deconvolute the complexity of factors regulating the glycosylation machinery could yield facile computational predictions of the glycosylation outcome.

### Software tools for predicting cellular glycosylation and the glycome

Here, we developed and make available an analysis framework that simplifies the process of predicting glycosylation capacity from RNA-seq data at both bulk and single cell levels. Using the R language, we have created a software package that can be integrated into other data analyses, that enables the prediction of both baseline glycosylation capacity, as well as prediction of differential glycosylation capacity from transcriptomic data. The software package takes advantage of calculated IQR ranges from our analyses and generates a visual representation of the glycosylation capacity given the input data. As we continue to update our predictor of glycosylation capacity, the software package will be updated. We have also made a simplified version of the functionality included in the package available on the website https://glyco.me.

### Conclusions and future applications

Our study provides a first attempt to use single cell RNA-seq transcriptomics data to study glycosylation and the glycome at the individual cell level. Although challenges clearly still exist we demonstrate that important information can be extracted with the current quality of data, and we present the Glycopacity software that enables investigators to use the approach more widely. We can use the obtained knowledge about regulatory hotspots of glycosylation and highly regulated glycogenes to validate and to develop and focus study designs into biological functions of glycosylation. Targeted proteomics, and when sensitivity allows single cell proteomics, can provide the next level of insights into the repertoire of glycosylation enzymes to further support the prediction of cellular glycosylation capacities and glycome outcomes. In summary, the atlas of glycosylation pathways and Glycopacity software provide tools toward single cell glycomics.

### Limitations of the study

The patterns and trends that are predicted in this study rely on the quality of the transcriptomic data that are used. Not all organs are sampled within both the organ-level and single cell datasets used, and not all cell types are represented. For single cell datasets, the cell types are computationally assigned, and thus may be erroneously labeled, or lack sufficient resolution to distinguish between cell types. Furthermore, the sensitivity of RNA-seq technologies to the low level of expression of glycosyltransferases place limitations on our ability to sensitively quantify glycogene expression levels.

## STAR★Methods

### Key resources table

**Table:** 

REAGENT or RESOURCE	SOURCE	IDENTIFIER
**Other**		

Glyco.me website	This paper	https://glyco.me
RGlycopacity R package	This paper	https://github.com/CopenhagenCenterForGlycomics/Rglycopacity https://doi.org/10.5281/zenodo.5793266
R scripts for analysis	This paper	https://github.com/CopenhagenCenterForGlycomics/Dworkin2022_iScience_figures https://doi.org/10.5281/zenodo.6481636
Panglao DB (mouse chromium 10x)	https://doi.org/10.1093/database/baz046	https://panglaodb.se/bulk.html
Tabula Sapiens V3.0	https://doi.org/10.6084/m9.figshare.14267219.v3	https://figshare.com/articles/dataset/Tabula_Sapiens_release_1_0/14267219/3; RRID:SCR_004328
scTransform datasets	This paper	Table S4
HEK293 mixture data	https://doi.org/10.1038/s41587-020-0534-z	https://singlecell.broadinstitute.org/single_cell/study/SCP426/single-cell-comparison-mixture-data; RRID:SCR_007073

### Resource availability

#### Lead contact

Further information and requests for resources and reagents should be directed to and will be fulfilled by the lead contact, Hiren J Joshi (joshi@sund.ku.dk).

#### Materials availability

N/A.

### Experimental model and subject details

N/A.

### Method details

#### Organ-level dataset description

We retrieved two independent bulk transcriptomic count datasets produced by the GTEx and TCGA consortiums, and hosted by the recount2 repository (Collado-Torres et al., 2017; GTEx Consortium, 2013). We selected all GTEx (all healthy), and the solid tissue normal samples (i.e., healthy) samples from TCGA for use in our analyses. Downloaded count data was first normalized to remove batch and sample biases using the trimmed mean of M values (TMM) method, available as part of the EdgeR software package (Robinson et al., 2010). Next, we used the same software library to calculate the counts per million (CPM) value, which is then log-transformed.

Since CPM values represent mRNA quantification as a proportion of the total reads mapped, and sequencing depth could vary between datasets, we decided to normalize the CPM values to a panel of housekeeping genes (shown in Figure S6), which makes the quantification of transcripts more robust to variation in sequencing depths. We surveyed candidate housekeeping genes by calculating benchmark correlation data between GTEx and TCGA CPMs for glycogenes from breast, lung, and prostate samples, rotating through the panel of candidate housekeeping genes. As all housekeeping genes performed similarly on the benchmark (Pearson correlation 0.86–0.89), we decided to make our normalization method robust to the absence of subsets of housekeeping genes, calculating the geometric mean of expression for all detectable housekeeping genes (CPM >0) in a sample, and then normalizing glycogene expression to this. We evaluated this normalization method using our benchmark, observing excellent performance (Pearson correlation 0.88, two-tailed t-test, p = 2.85E-180, n = 587, Figure S6).

#### Single cell dataset description

The choice of single cell dataset was important when performing the analysis of single cell data. The criteria we used to determine which dataset to use were: a) That datasets are uniformly processed and annotated, b) Datasets include tissue and organ diversity so that we can sample as much diversity in glycogene expression variation as possible. The Tabula Sapiens repository of datasets is a high-quality dataset with cell types that have been both manually annotated by experts and automatically annotated by the PopularVote annotation pipeline (Consortium, 2022). PanglaoDB (Franzén et al., 2019) is an online repository of human and mouse single cell datasets originating from publicly available NCBI sequence read archives (SRA) that have been uniformly processed by the alona (Franzén and Björkegren, 2020) pipeline. Metadata associated with each dataset that was pertinent to our analyses include the organism, tissue, per-cell cell type annotation, barcode and t-SNE cluster number. The bulk of the data is in the form of an *n* cell barcode x *m* unique molecular identifier (umi) raw count matrix. Only Chromium10x datasets were used in PanglaoDB analyses, whereas both Chromium10x and Smartseq2 datasets were used in Tabula Sapiens analyses.

#### Validation that pseudo-bulk calculation does not introduce biases

As an additional check to ensure that normalization and pseudo-bulk quantitation calculations performed on the single cell data did not introduce any biases, we performed a separate estimation of count values using count data from PanglaoDB, Human Cell Atlas, EBI expression atlas, and 10x genomics (Franzén et al., 2019; Papatheodorou et al., 2020; Regev et al., 2017) (Data sources listed in Table S4). A variance stabilizing transformation with default parameters was performed on each raw count matrix using the *vst* function of the scTransform package version 0.3.2 (Hafemeister and Satija, 2019). Multiple organ systems and cell types are represented by selected datasets, providing a good overlap with the set of annotated systems present across clusters.

We did not integrate clusters of single cells from different experiments for this analysis, so different clusters may represent the same cell types from different tissue samples. We believe these duplicates will not materially affect the conclusions of this analysis, and further analyses could make use of recent algorithmic advances to solve this problem (Stuart et al., 2019). The labeling of cell type within the PanglaoDB pipeline cannot ensure accuracy of labels, so 2822 mouse clusters lack cell type annotation, and the cell type annotation may be incorrect in specific cases. We have limited the risks of incorrect annotation within our analysis by first dropping data where cell type annotation is missing, and then only relying on specific cell type annotation for intestinal and kidney cells in mice (Figure 5).

#### Single cell data preparation

Raw count data from both Tabula Sapiens and PanglaoDB were prepared for analysis. The Tabula Sapiens data required no filtering because of sufficient pre-processing. PanglaoDB raw count matrices were filtered to include only those clusters annotated with a defined cell type (i.e., no “unknown” cluster cell type annotations). Then using Scanpy version 1.7 (Wolf et al., 2018), the library associated with each cell barcode was normalized so that the total counts for each cell is the same (set to 10,000 counts as per the defaults for *normalize_total*). Finally values were log1p transformed using the Scanpy *log1p* function. To stabilize values and remove outliers for each library, non-zero umi counts outside of the 10–90^th^ percentile range of all detectable counts in the library were masked by setting to a null value.

#### Calculation of pseudo-bulk quantitation for a cluster of cells

To group single cells together into a cluster, we made use of the supplied cluster annotations from our source datasets. We then treated this cluster of cells as a pseudo-bulk, calculating the trimmed mean of the umi counts for each gene with detectable transcripts in the cluster (dropping non-zero counts outside of the 10–90^th^ percentile range of values for the gene in the cluster).

#### Cluster filtering criteria

The individual clusters of cells that were used for further analysis were selected by applying a quality control filter to the complete set. Each cluster must have a tissue/organ and cell type annotation other than ‘unknown’, such that we could consider each cluster as representative of a unique cell identity (i.e., a cell type). Furthermore, we required that each cluster was made up of at least 200 cells, which we estimated as the minimum number of cells to reliably estimate glycogene expression. To our knowledge, the N-linked glycosylation pathway is active in all mammalian cells, and so we ensured that a set of genes responsible for biosynthesis of the N-linked precursor (DPAGT1, ALG2, ALG13 or ALG14) were expressed above a minimal cut-off (see below for cut-off calculation). A total of 229 human and 2317 mouse clusters successfully passed these filters.

#### Normalization on housekeeping genes

As with the organ-level data, we needed to normalize the transcript quantitation for each pseudo-bulk that was collected with potentially different sequencing depth. Using the same selection of housekeeping genes as used for the organ level normalization, we normalized the pseudo-bulk quantitation values to the average pseudo-bulk quantitation for the 21 housekeeping genes (Figure S6), both of which were log-transformed so that they were comparable to the organ-level log-transformed CPMs.

#### Centered log ratio transform

We additionally introduced the use of the centered log ratio (CLR) transformation to the pseudo-bulk quantitation values as a method to determine which glycogenes were highly expressed in individual cell types without reference to the IQRs as calculated by bulk data. We calculated the CLR directly on the pseudo-bulk quantitation values after trimmed mean calculation and filtering, using the *clr* method of the compositions package (van den Boogaart and Tolosana-Delgado, 2008).

#### Calculation for cut-off and thresholds for single cell data

As we could not use a simple 1 CPM cut-off as per the organ level RNA-seq data, we developed a mathematical model to estimate the minimal pseudo-bulk quantitation that should be observed for a glycogene for it to be considered as expressed. We based our model on analysis of a dataset of paired bulk RNA-seq and single cell RNA-seq from human HEK293 cell lines (Ding et al., 2020). We modeled the relationship between the transcript quantitation in the bulk data and the single cell data. Our model relates the TPM values from bulk RNA-seq to the calculated pseudo-bulk quantitations as calculated from the single cell data, for varying cluster sizes (which we simulated by sampling clusters from the full HEK293 single cell dataset) (Figure S4A). Correlations were the highest when using the most number of cells in a simulated cluster (Spearmans correlation coefficient, r_s_ = 0.898, one-tailed t-test, p = 2.2E-16, n = 34187), and the patterns were similar when examining glycogenes only (Figure S4B). Using our model, we can read the pseudo-bulk quantitation cut-off, and the variation in this value (i.e., cluster size dependent SD) from the intersection with a one TPM cut-off. Given a single pseudo-bulk quantitation value and number of cells in the cluster that comprise the pseudo-bulk, we can test whether this value passes the cut-off, using a two-sided one sample t-test on the pseudo-bulk quantitation cut-off and cluster size appropriate SD.

The model was built using a series of seven linear regression models, for different simulated cluster sizes (n = 50, 100, 200, 400, 600, 800, 1000). The linear regressions modeled the relationships between the average pseudo-bulk quantitation values for ∼200 glycogenes in 100 clusters formed by sampling all cells, and their corresponding TPM values. For all seven models, the cut-off at one TPM was invariant to cluster size, so we used the average cut-off as calculated by all models for this analysis (0.0054). The variance of the cut-offs are sensitive to cluster size, so we calculated the variance in pseudo-bulk quantitation values for 224 glycogenes in 100 sampled clusters, and fit a curve to describe the relationship between cluster size and pseudo-bulk quantitation variation at one TPM (Figure S4C).

### Quantification and statistical analysis

All statistical details of experiments, including the statistical tests used, exact value of n, and what n represents can be found in results, methods and supplementary figure legends. Significance was defined as p less than or equal to 0.05.

### Additional resources

#### R package glycopacity

The Glycopacity R package enables prediction of baseline glycosylation capacity and comparison of differential glycosylation capacity from transcriptomic data. Using the methods with a set of glycogenes and their corresponding pseudo-bulk quantitation values, the baseline glycosylation capacity of each cluster can be predicted using the previously established cut-off and threshold model. Baseline and differential glycosylation capacity can be visualized on the rainbow depiction as overlay heatmaps to aid in comprehension of predicted features.

Methods for comparing differential glycosylation capacities are generalizable across quantitation methodologies, allowing for interchangeable comparison of user-supplied single cell and bulk datasets with precomputed and normalized quantiles from the GTEx, TCGA, Tabula Sapiens, and PanglaoDB datasets used in this study.

The package is available for download (URLs in the Key resources table) or via the main website at https://glyco.me.

#### Glyco.me website

We have implemented the Glycopacity algorithm at our in silico glycomics analysis portal at https://glyco.me.

## Data Availability

• This paper analyses existing, publicly available data. The accession numbers for the datasets are listed in the Key resources table. • All original code (i.e., scripts to produce figures and a R package) has been deposited at Zenodo and is publicly available as of the date of publication. Accession numbers and DOIs are listed in the Key resources table. • Any additional information required to reanalyze the data reported in this paper is available from the Lead contact upon request.

## Supplemental information

Document S1. Figures S1–S6

Table S1. Samples and organ info, related to Figure 1

Table S2. Celltype counts, related to Figure 1

Table S3. Celltype glycosylation capacities, related to Figure 5

Table S4. SRA descriptions of data sources, related to Figure 3
