# Supplementary material for: Applying transcriptomics to studyglycosylation at the cell type level
Source: iScience. 2022 May 18;25(6):104419. doi: 10.1016/j.isci.2022.104419 (PMC9156939; doi:10.1016/j.isci.2022.104419)

iScience, Volume *25*

Supplemental information

Applying transcriptomics to study glycosylation at the cell type level

Leo Alexander Dworkin, Henrik Clausen, and Hiren Jitendra Joshi

Supplemental Figure Legends

Figure S1. The rainbow depiction of glycogene pathways, Related to Figure 1. This representation of glycosylation pathways is designed to give a global view of all the pathways and the outcomes of the glycosylation process, alongside the assigned roles for glycogenes in the process (adapted from (Schjoldager et al., 2020)). The major pathways are arranged along the vertical axis, and glycogenes are furthermore split into groups indicating participation in the initiation, core extension, elongation and capping steps of glycosylation. This same grouping also separates out genes that are classified into only single pathways, and those that potentially work across multiple pathways – giving a visual indication that the elongation and capping steps are encoded by genes that are not necessarily pathway specific. Glycan symbols are drawn according to the SNFG format (Neelamegham et al., 2019).

Figure S2. Rainbow figures with hotspots of regulation for bulk and single-cell RNA-seq data, Related to Figure 2 and Figure 3. Complete heatmaps from which Figure 2 and Figure 3 are extracted.

Figure S3. Complete comparisons of transcriptomic data between the single cell and organ level, Related to Figure 2. A) Histograms of the distributions of log-transformed and housekeeping gene-normalised bulk CPMs and single cell pseudo-bulks for 224 glycogenes. Boxplots highlighting the bulk IQR fold change of GTEx values greater than a 1 CPM cut-off accompany each gene. Histograms of bulk values from GTEx (dark grey) and TCGA (light grey) are plotted above the density curves of pseudo-bulk quantitation values from Tabula Sapiens (red) and PanglaoDB mouse (blue). Mouse cell types with unknown annotation are given a light blue colour for the density curves. Both bulk GTEx (dark green) and TCGA (light green) and single cell values less than a 1 CPM cut-off are indicated via inclusion of the predicted single cell expression cut-off (black vertical line). Estimation of gene expression levels as calculated by scTransform (grey density) are included for reference. B) Pearson correlation between gene specific IQRs of normalised bulk CPMs and single cell pseudo-bulk quantitation values. Single cell and bulk IQRs are computed before (red) and after (blue) filtering out pseudo-bulk quantitation values that fail to pass the minimal expression cut-off, or have a bulk CPM less than 1. This filtering results in greater correlation, as indicated by tighter adherence of each point to the identity function, and a decrease in the number of clusters to compute the IQR for a gene (saturation). C) Pearson correlation between gene specific means of normalised bulk CPMs and single cell pseudo-bulk quantitation values. Interpretation is same as in B. In B and C, filtered TCGA bulk CPMs and single cell pseudo-bulk quantitation values have highest correlation between gene IQRs (Pearson correlation coefficient, $r = 0.53$, two-tailed t-test, $p = 1.30E-17$, $n = 222$) and means (Pearson correlation coefficient, $r = 0.71$, two-tailed t-test, $p = 1.85E-34$, $n = 222$). D) Pearson correlation between gene specific IQRs of normalised bulk CPMs and single cell pseudo-bulk within bulk CPM delimited intervals. Correlation between bulk CPMs and single cell pseudo-bulk quantitation values is highest and with greatest significance (Pearson correlation coefficient, $r = 0.43$, two-tailed t-test, $p = 2.71E-06$, $n = 108$) within the bulk IQR interval of 0.5-1.

Figure S4. Modelling minimal expression capacity, Related to Figure 3. Linear modelling of A) all gene, or B) glycogene quantitation values in paired bulk and single cell replicates. Plotted points and fit in all panels, and top spearman correlation in all panel tables use all cells of single cell replicate to compute pseudo-bulk quantitation values. Tables in each panel display three spearman correlation averages and standard deviations generated from sample populations (n = 100), each population differing by the number of cells in a sample used to compute pseudo-bulk quantitation values (number of cells = 86, 215, 430). Standard deviation of spearman correlation increases with decreasing number of cells for glycogenes. C) The cut-off and threshold model used for prediction of minimal expression capacity in this study.

Figure S5. Overview of proportions of cells expressing glycogenes, Related to Figure 3. A) Histogram of the number of glycogenes with pseudo-bulk quantitation values passing the minimal expression cut-off in some percent of clusters. Bars are coloured by the percent of clusters in which the cut-off was not passed B) Percent of glycogenes passing minimal expression cut-off in some percent of clusters. Similar curves can be seen for human and mouse clusters from Tabula Sapiens (blue) and PanglaoDB (red).

Figure S6. Housekeeping gene benchmarks, Related to Figure 2 and Figure 3. Each scatterplot consists of mean tissue natural log1p transformed CPMs of 195 glycogenes that are shared between GTEx and TCGA in three tissues (red = breast, green = lung, blue = prostate) and that have been normalised to a housekeeping gene (n = 21), the geometric mean of all housekeeping genes (panel), or DPAGT1 (inset). Pearson correlation between normalised values of GTEx and TCGA remains strong ($r \geq 0.86$) regardless of the housekeeping gene chosen for normalisation. As CPMs normalised on mean housekeeping gene expression (Pearson correlation coefficient, r = 0.88, two-tailed t-test, p = 2.85E-180, n = 587) correlate similarly to data normalised on DPAGT1, this normalised expression is used in downstream analyses.

Supplemental Figure 1 - The rainbow depiction of glycogene pathways

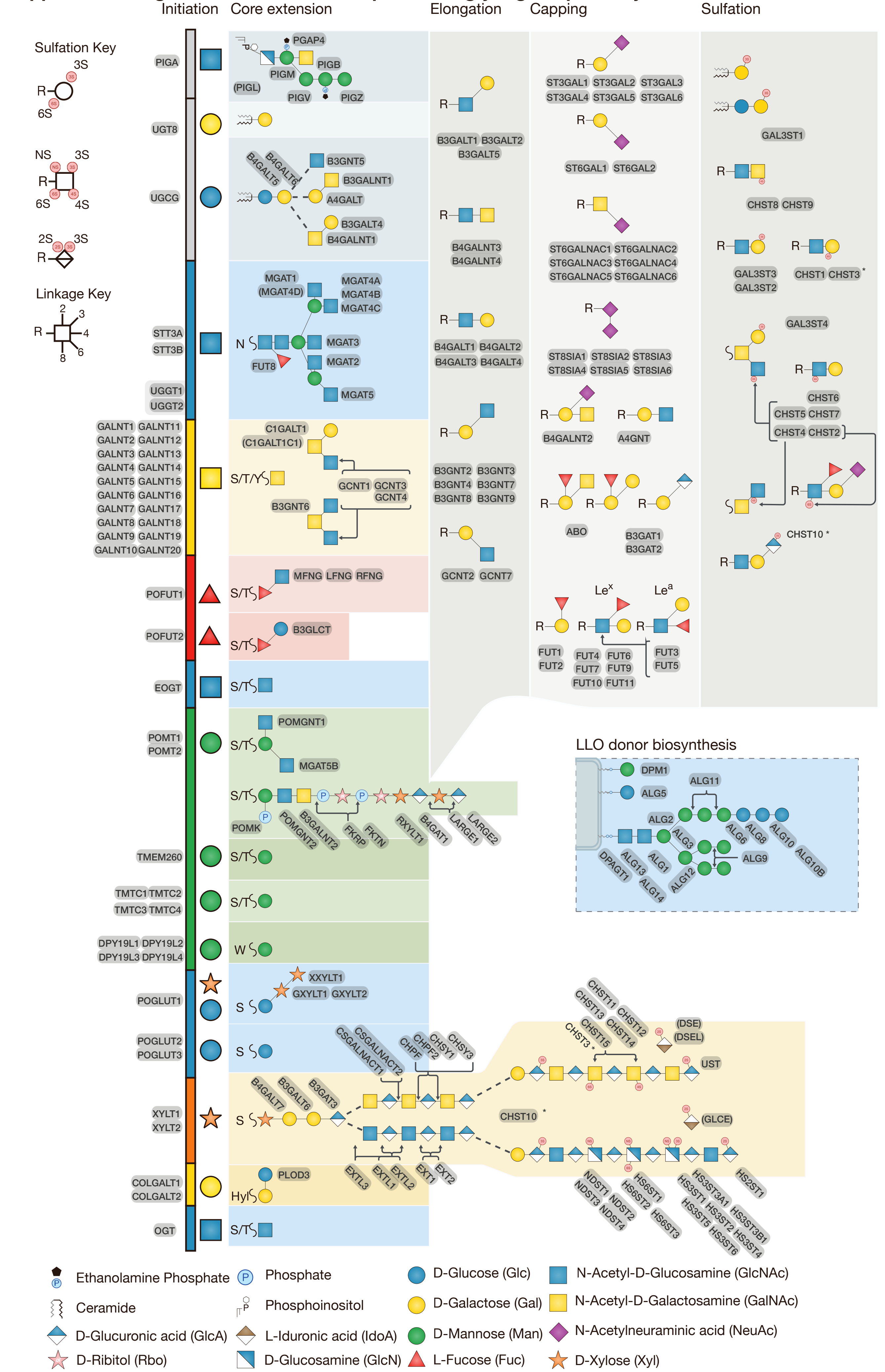

Supplemental Figure 2 - Rainbow figures with hotspots of regulation for bulk and single-cell RNA-seq data

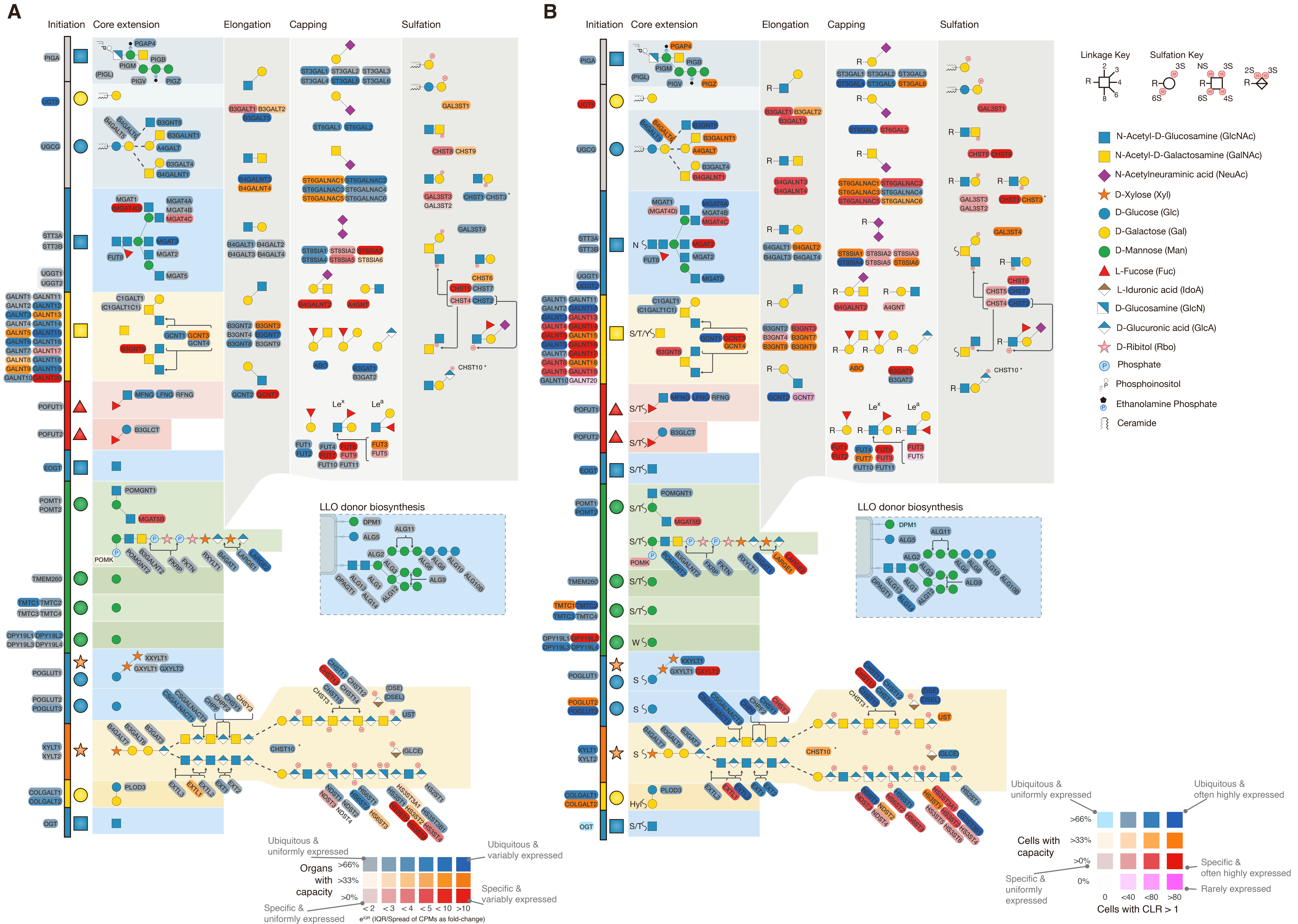

Supplemental Figure 3 - Complete comparisons of transcriptomic data between the single cell and organ level

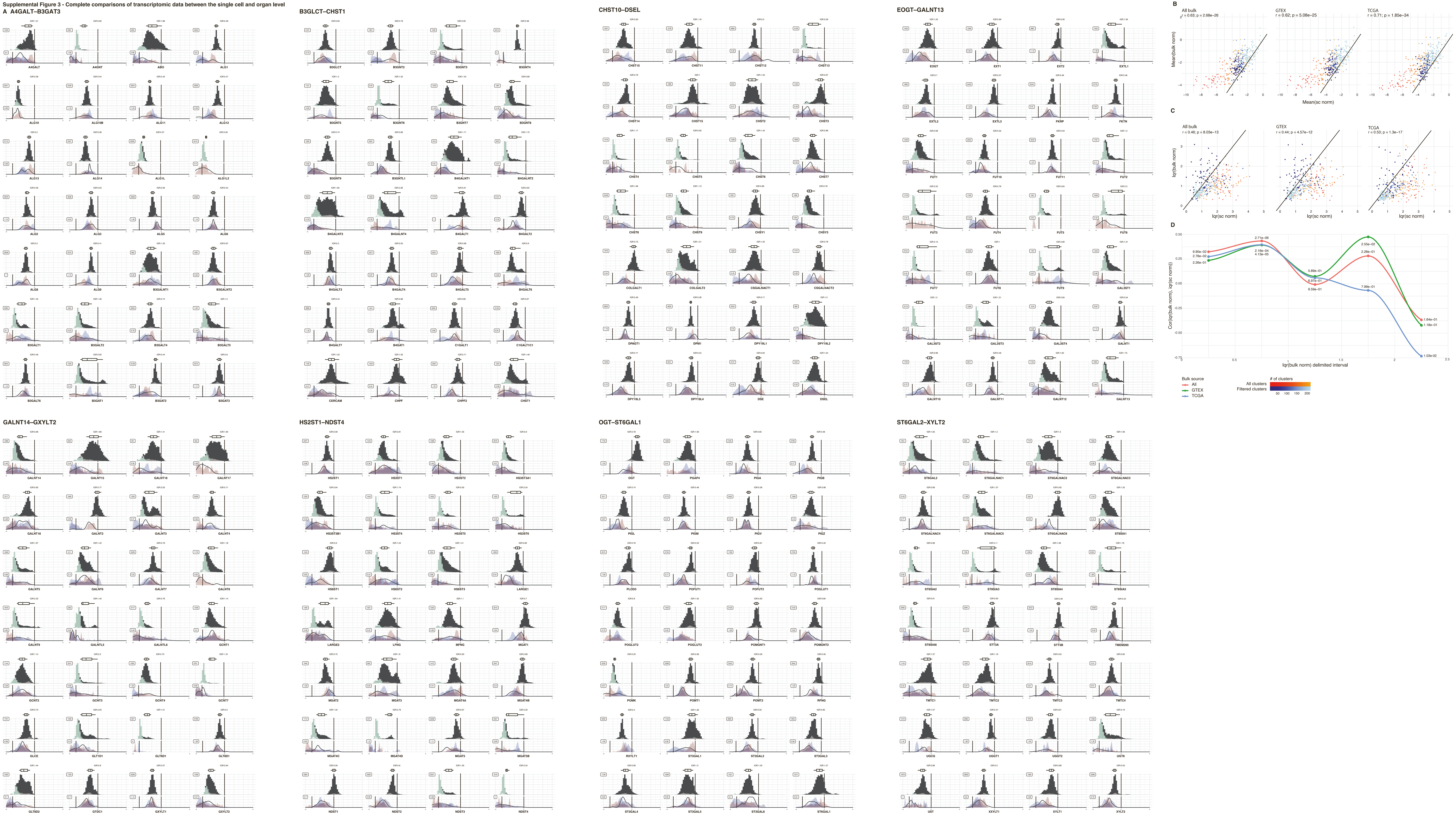

Supplemental Figure 4 - Modelling minimal expression capacity

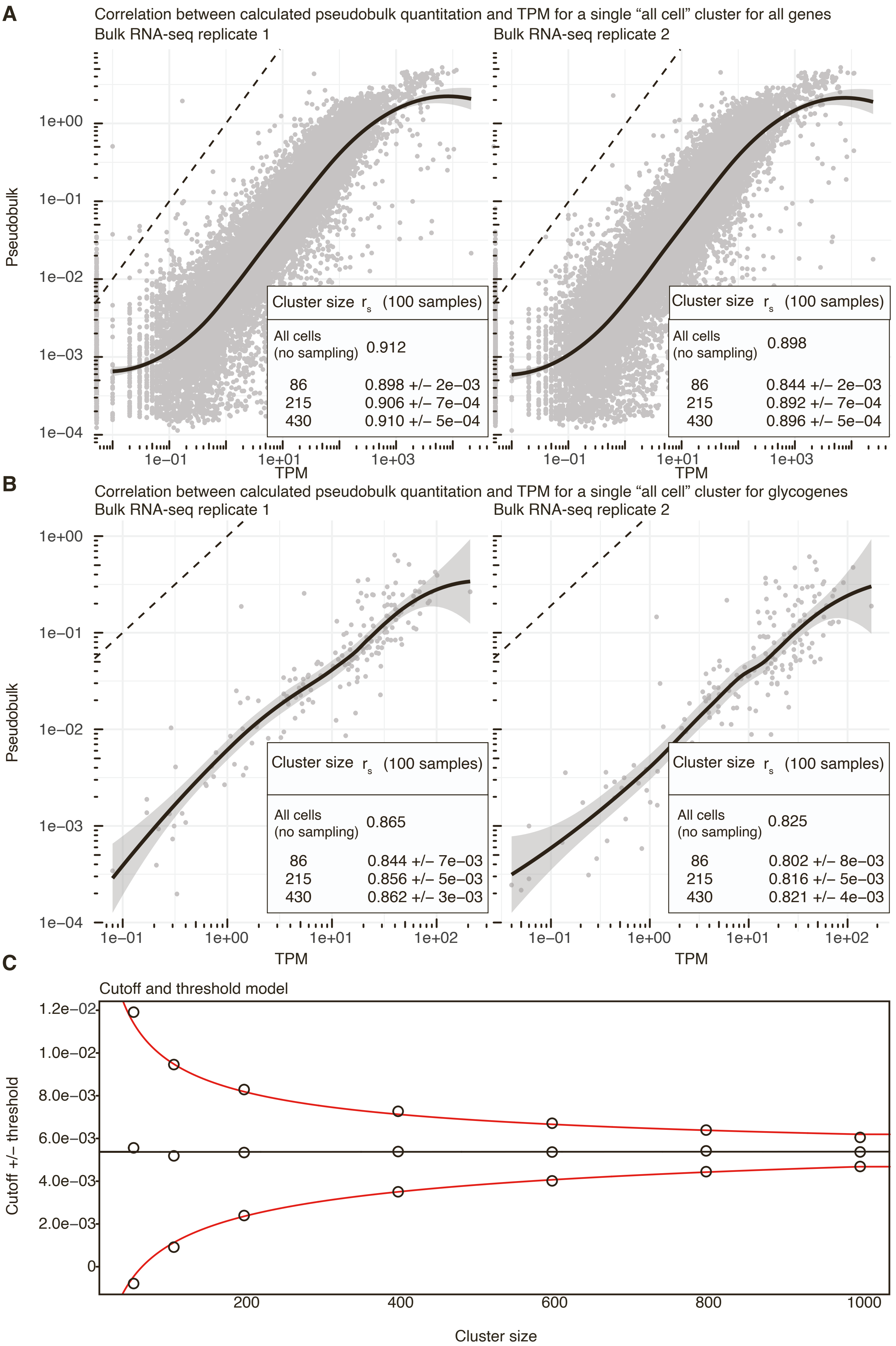

Supplemental Figure 5 - Overview of proportions of cells expressing glycogenes

A

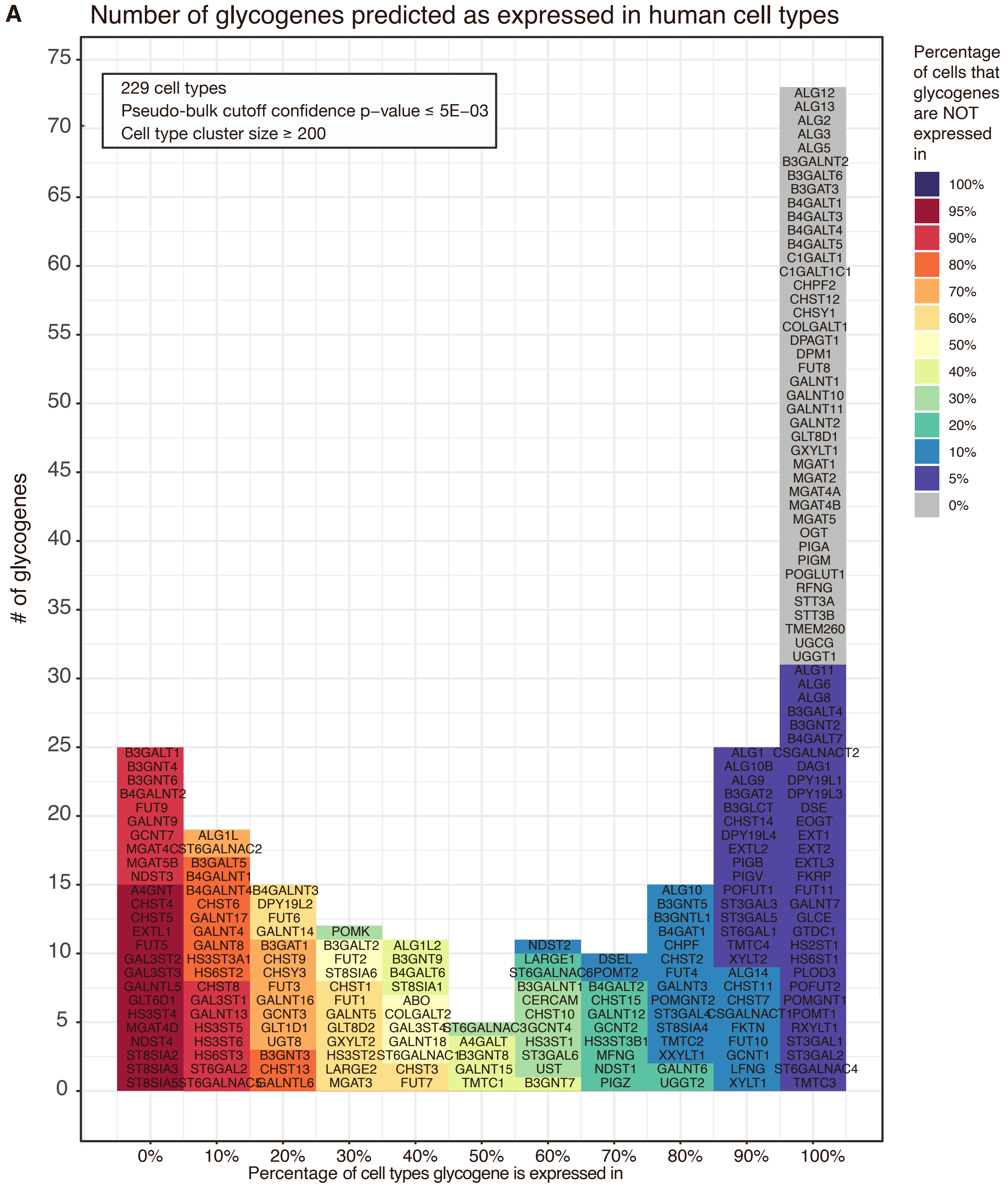

B

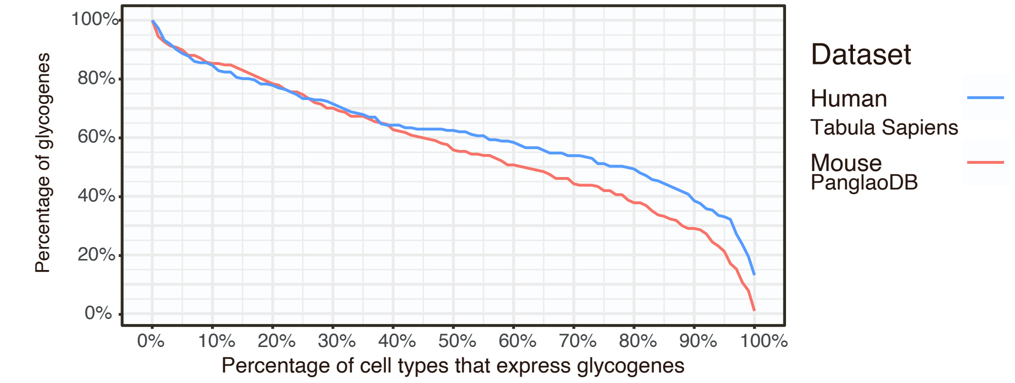

Supplemental Figure 6 - Housekeeping gene benchmarks

Mean relative expression of 195 GTfs normalized to 21 housekeeping genes, all housekeeping genes, and DPAGT1

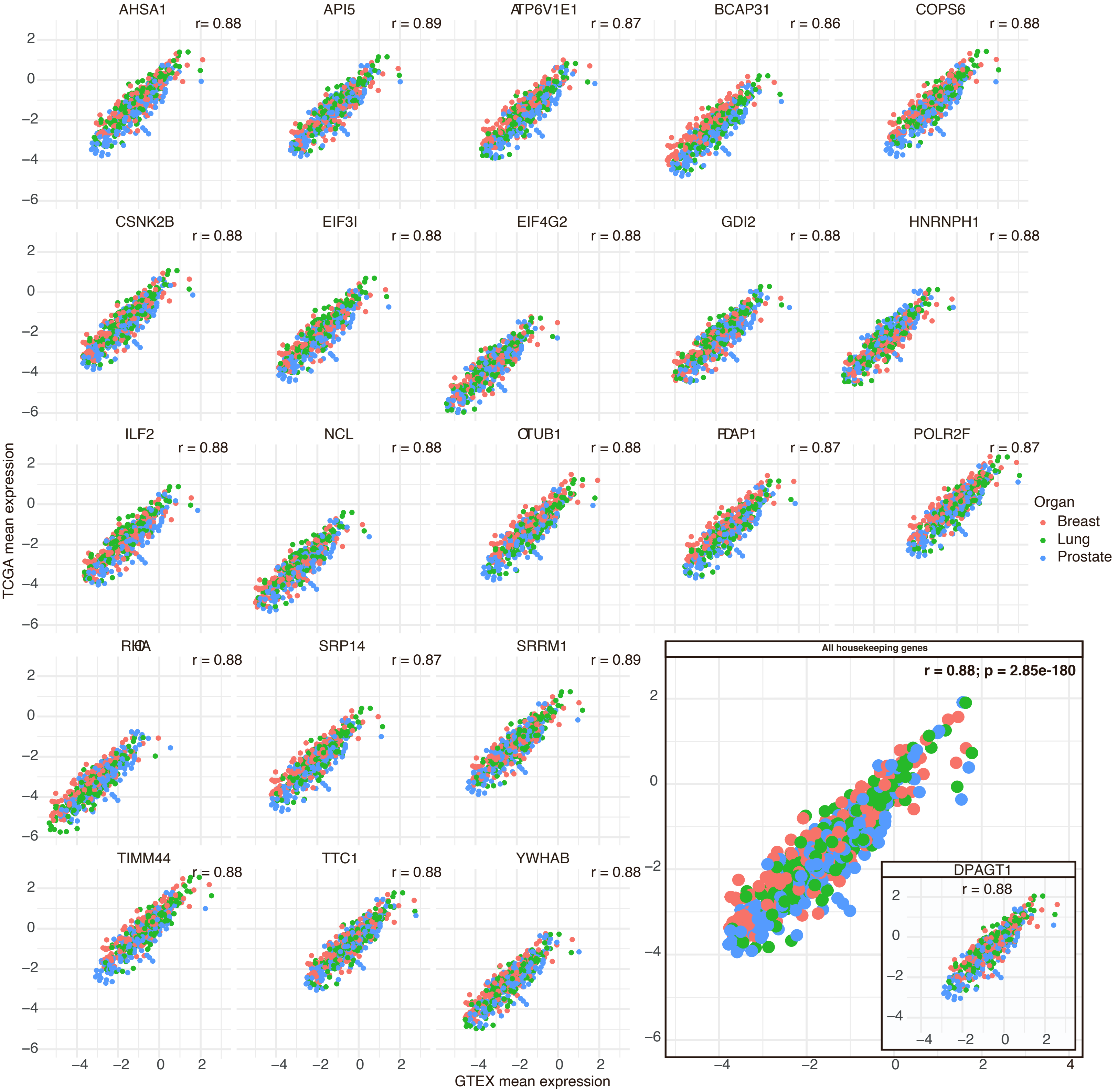

Supplement: Document S1. Figures S1–S6 [file mmc1.pdf]
